# Health-related quality of life in breast cancer patients: A bibliographic review of the literature from 1974 to 2007

**DOI:** 10.1186/1756-9966-27-32

**Published:** 2008-08-29

**Authors:** Ali Montazeri

**Affiliations:** 1 Iranian Institute for Health Sciences Research (IHSR), Tehran, Iran; 2 Iranian Centre for Breast Cancer (ICBC), Tehran, Iran

## Abstract

**Background:**

Quality of life in patients with breast cancer is an important outcome. This paper presents an extensive overview on the topic ranging from descriptive findings to clinical trials.

**Methods:**

This was a bibliographic review of the literature covering all full publications that appeared in English language biomedical journals between 1974 and 2007. The search strategy included a combination of key words 'quality of life' and 'breast cancer' or 'breast carcinoma' in titles. A total of 971 citations were identified and after exclusion of duplicates, the abstracts of 606 citations were reviewed. Of these, meetings abstracts, editorials, brief commentaries, letters, errata and dissertation abstracts and papers that appeared online and were indexed ahead of publication were also excluded. The remaining 477 papers were examined. The major findings are summarized and presented under several headings: instruments used, validation studies, measurement issues, surgical treatment, systemic therapies, quality of life as predictor of survival, psychological distress, supportive care, symptoms and sexual functioning.

**Results:**

*Instruments*-Several valid instruments were used to measure quality of life in breast cancer patients. The European Organization for Research and Treatment of Cancer Core Cancer Quality of Life Questionnaire (EORTC QLQ-C30) and its breast cancer specific complementary measure (EORTC QLQ-BR23) and the Functional Assessment Chronic Illness Therapy General questionnaire (FACIT-G) and its breast cancer module (FACIT-B) were found to be the most common and well developed instruments to measure quality of life in breast cancer patients. *Surgery*-different surgical procedures led to relatively similar results in terms of quality of life assessments, although mastectomy patients compared to conserving surgery patients usually reported a lower body image and sexual functioning. *Systemic therapies*-almost all studies indicated that breast cancer patients receiving chemotherapy might experience several side-effects and symptoms that negatively affect their quality of life. Adjuvant hormonal therapies also were found to have similar negative impact on quality of life, although in general they were associated with improved survival. *Quality of life as predictor of survival*-similar to known medical factors, quality of life data in metastatic breast cancer patients was found to be prognostic and predictive of survival time. *Psychological distress*-anxiety and depression were found to be common among breast cancer patients even years after the disease diagnosis and treatment. Psychological factors also were found to predict subsequent quality of life or even overall survival in breast cancer patients. *Supportive care*-clinical treatments to control emesis, or interventions such as counseling, providing social support and exercise could improve quality of life. *Symptoms*-Pain, fatigue, arm morbidity and postmenopausal symptoms were among the most common symptoms reported by breast cancer patients. As recommended, recognition and management of these symptoms is an important issue since such symptoms impair health-related quality of life. *Sexual functioning*-breast cancer patients especially younger patients suffer from poor sexual functioning that negatively affect quality of life.

**Conclusion:**

There was quite an extensive body of the literature on quality of life in breast cancer patients. These papers have made a considerable contribution to improving breast cancer care, although their exact benefit was hard to define. However, quality of life data provided scientific evidence for clinical decision-making and conveyed helpful information concerning breast cancer patients' experiences during the course of the disease diagnosis, treatment, disease-free survival time, and recurrences; otherwise finding patient-centered solutions for evidence-based selection of optimal treatments, psychosocial interventions, patient-physician communications, allocation of resources, and indicating research priorities were impossible. It seems that more qualitative research is needed for a better understanding of the topic. In addition, issues related to the disease, its treatment side effects and symptoms, and sexual functioning should receive more attention when studying quality of life in breast cancer patients.

## Background

Health-related quality of life is now considered an important endpoint in cancer clinical trials. It has been shown that assessing quality of life in cancer patients could contribute to improved treatment and could even be as prognostic as medical factors could be prognostic [1-4]. Above all, studies of quality of life can further indicate the directions needed for more efficient treatment of cancer patients. Among the quality of life studies in cancer patients, breast cancer has received most attention for several reasons. First, the number of women with breast cancer is increasing. It has been reported that each year over 1.1 million women worldwide are diagnosed with breast cancer and 410,000 die from the disease [5]. Secondly, early detection and treatment of breast cancer have improved and survivors now live longer, so studying quality of life in this context is important. Thirdly, breast cancer affects women's identities and therefore studying quality of life for those who lose their breasts is vital. In addition, it is believed that females play important roles as partners, wives, and mothers within any family. Thus, when a woman develops breast cancer, all members of family might develop some sort of illnesses. In fact, breast cancer is a family disease. Other reasons could be added, but overall it is crucial to recognize that with increasing improvements in medicine and medical practice during recent years studying quality of life for any cancer, for any anatomical site and for either gender is considered highly relevant. A descriptive study of the published papers (230 articles) on non-biomedical outcomes (quality of life, preferences, satisfaction and economics) in breast cancer patients, covering the literature from 1990 to 2000, found that the most frequently reported outcomes were health-related quality of life (54%), followed by economic analyses (38%), and patient satisfaction (14%). Only 9% measured patient preferences [6].

Over the past 10 years, much clinical effort has been expended in the treatment of breast cancer in order to improve survival. Now the question is: to what extent have studies of quality of life in breast cancer patients added to our information or contributed to improved outcomes in breast cancer care? This is very difficult to answer, but it is possible to try to investigate the contribution of quality of life studies to breast cancer care as a whole. There are several useful review papers on quality of life in breast cancer patients. However, most published papers have either been overviews or systematic literature searches with very focused objectives. The aim of this review is to collect and examine all literature published since the topic first appeared in English language biomedical journals. It is hoped that this extensive review may contribute to existing knowledge, help both researchers and clinicians to have a better profile on the topic, and consequently aid in improving quality of life in breast cancer patients.

## Methods

As part of a study on quality of life in breast cancer patients, an extensive literature search was carried out using MEDLINE, EMBASE, the Science Citation Index (ISI), the Cumulative Index to Nursing and Allied Health Literature (CINAHL), the PsycINFO, the Allied and Complementary Medicine (AMED), and Global Health databases. The intention was to review all full publications that have been appeared in English language biomedical journals between 1974 and 2007. The year 1974 was chosen because the first study on quality of life in breast cancer patients was published then. The search strategy included the combination of key words 'quality of life' and 'breast cancer' or 'breast carcinoma' in titles of publications. It was though that this might help to focus the investigation. It provided the initial database for the review. The initial search was carried out in early 2006 and updated twice in 2006, twice at the end of January and December 2007, and once for a final check in April 2008.

## Results

### Statistics

A total of 971 citations were identified and after exclusion of duplicates, the abstracts of 606 citations were reviewed. Of these, meetings abstracts, editorials, brief commentaries, letters, errata and dissertation abstracts and papers that appeared online and were indexed ahead of publication were also excluded. The remaining 477 papers were examined in this bibliographic review. The statistics are shown in Table 1 and a chronological list of all papers is available [Additional file 1]. Here, the major findings are summarized and presented under the following headings.

**Table 1:** Number of citations by year of publication (1974–2007)

**Year**	**Breast cancer**	**Quality of life**	BC+QOL*	Papers reviewed**
1974	246	13	1	1
1975	312	23	0	0
1976	358	34	1	1
1977	522	27	0	0
1978	527	33	0	0
1979	489	34	0	0
1980	662	36	1	1
1981	634	45	1	0
1982	647	71	1	1
1983	661	89	2	2
1984	830	73	0	0
1985	844	97	2	2
1986	920	134	1	1
1987	961	211	2	2
1988	1125	223	2	2
1989	1333	294	2	2
1990	1470	422	7	6
1991	1423	394	8	7
1992	1805	603	8	8
1993	2088	641	18	17
1994	2342	747	16	15
1995	2444	948	11	10
1996	2926	1422	16	15
1997	3249	1756	19	16
1998	3597	2049	29	25
1999	3872	2457	39	30
2000	5026	2639	37	30
2001	5206	2985	34	27
2002	5720	3233	42	26
2003	6441	3900	38	31
2004	7422	4811	74	47
2005	7862	5276	73	53
2006	7021	4592	63	48
2007	4641	2207	58	51

**Total**	85626	42519	606	477

* Excluding duplicates and papers that appeared online and indexed ahead of publication.

** Excluding all meetings abstracts, editorials, brief commentaries, letters, replies, erratum, and dissertation abstracts. For all citations see Additional file 1.

### Reviews

There were several review papers. These were divided into two categories: overviews [7-26], and systematic reviews [27-35]. Whilst there were quite significant numbers of commentaries, some brief, a few systematic reviews with focused objectives were also identified. These are summarized in Tables 2 and 3. Both overviews and systematic reviews touched interesting topics pointed to helpful comments and findings among published papers. For instance, a paper by Rozenberg et al. [26] highlighted that most women affected by breast cancer will not die from it but from other diseases, owing to recent improvements in treatment. They also pointed out that women with breast cancer and three or more co-morbid conditions have a 20-fold higher rate of mortality from causes other than breast cancer and a 4-fold higher rate of all-cause mortality when compared with patients who have none.

**Table 2:** A list of some overview papers on quality of life in breast cancer patients (1974–2007)

**Author(s) [Ref.]**	**Year**	**Main focus**	**Conclusion(s)**
McEvoy and McCorkle [7]	1990	QOL in advanced breast cancer	Efforts to manage advanced breast cancer must include both current medical therapies and attention to the critical factors associated with enhancing their QOL.

Kiebert et al. [8]	1991	Impact of breast conserving surgery vs. mastectomy on QOL	There were no substantial differences between the two treatment modalities except for body image and sexual functioning in favor of breast conserving surgery.

Aarenson [9]	1993	Assessments of QOL and benefits from adjuvant therapies	Adjuvant therapies could improve QOL in breast cancer patients.

Bryson and Plosker [10]	1993	Tamoxifen as adjuvant therapy	Tamoxifen has a low cost-utility ratio in postmenopausal women with node-positive, estrogen receptor-positive breast cancer.

Stefanek [11]	1994	QOL research, provider-patient communication, and psychological distress of spouses and other relatives of breast cancer patients	This review summarizes and critiques publications in three identified areas.

Ganz [12]	1994	Review of various approaches to the measurement of QOL, the important QOL issues in the treatment of breast cancer, and what is known about QOL of older women with breast cancer	Ongoing and future research using newer approaches to QOL assessment should provide additional information on this important topic.

Osoba [13]	1994	QOL as a treatment endpoint	Advances in understanding HRQOL in metastatic breast cancer will aid the development of rational treatment policies.

Carlson [14]	1998	QOL in metastatic breast cancer	Clinician must balance anti-tumor activity, performance status, and the usual toxicity measures as surrogates for QOL associated with each specific therapy.

Leedham and Ganz [15]	1999	Psychological concerns and mental health	Psychological concerns and mental health are important issues for breast cancer patients and should be recognized and treated when necessary.

Rustoen and Begnum [16]	2000	Nursing practice	Nurses play an important role in meeting the needs of breast cancer patients.

Shapiro et al. [17]	2001	Relationship between psychosocial variables and QOL	A broader, more integrative framework that includes psychosocial factors is needed to evaluate breast cancer consequences.

Partridge et al. [18]	2001	QOL before, during and after high-dose chemotherapy	Resulting transient impaired overall QOL with subsequent improvement over time.

Kurtz and Dufour [19]	2002	QOL in older patients with metastatic disease receiving either standard treatment or new drugs	Aromatase inhibitors (such as taxanes and orally administered chemotherapy) provide similar or a better QOL as compared to first line endocrine therapy with tamoxifen.

Costantino [20]	2002	Hormonal treatments in metastatic breast cancer patients	QOL data is useful for both clinicians and patients in evaluating treatment options and developing treatment strategies.

Fallowfield [21]	2004	Hormonal therapies	Tolerability profiles of available treatment options are highlighted.

Sammarco [22]	2004	QOL of older breast cancer patients	Outpatient and long-term care should become a key setting for implementation of QOL interventions for women with breast cancer.

Knobf [23]	2006	Endocrine effects of adjuvant therapy in younger survivors	Causes premature menopause that is associated with poorer QOL, decreased sexual functioning, menopausal symptom distress, psychosocial distress related to infertility, and infertility.

Kayl and Meyers [24]	2006	Side effects of chemotherapy	QOL issues may help to guide patient-care decision.

Diel [25]	2007	Effectiveness of bisphosphonates on bone pain and quality of life in breast cancer patients with metastatic bone disease	Clinical trial data demonstrate that bisphosphonates offer significant and sustained relief from bone pain and can also improve quality of life in patients with metastatic breast cancer. New treatment schedules using high dose bisphosphonates can offer rapid relief of acute, and severe bone pain.

Rozenberg et al. [26]	2007	Co-morbid conditions and breast cancer	Women with breast cancer and three or more co-morbid conditions have a 20-fold higher rate of mortality from causes other than breast cancer and a 4-fold higher rate of all-cause mortality when compared with patients who have none.

**Table 3:** A list of systematic reviews on different aspects of quality of life in breast cancer patients (1974–2006)

**Author(s) [Ref.]**	**Year**	**Main focus**	**Conclusion(s)**
Irwig and Bennetts [27]	1997	A systematic review of quality of life after breast conservation or mastectomy	Apart body image it is unclear whether breast conservation or mastectomy results in better psychosocial outcomes.

Bottomley and Therasse [28]	2002	Systemic therapy (chemotherapy, hormonal therapy, or biological therapy) in advanced breast cancer (1995–2001)	QOL data provide invaluable insights into the treatment and care of patients.

Shimozuma et al. [29]	2002	Systematic overview of the literature (1982–1999)	To date there have been almost no appropriate systematic overviews or guidelines issued for QOL assessment studies related to breast cancer.

Goodwin et al. [30]	2003	Randomized clinical trials of treatment (review of literature from 1980–2001)	Until results of ongoing trials in breast cancer are available, caution is recommended in initiating new QOL studies unless treatment equivalency is expected or unless unique or specific issues can be addressed.

Rietman et al. [31]	2003	Late morbidity of breast cancer (review of literature from 1980 to 2000)	Significant relationship between late morbidity and restrictions of daily activities and poorer QOL was reported.

Payne et al. [32]	2003	Racial disparities in the palliative care for African-American (review of literature from 1985 to 2000)	Differences in treatment patterns, pain management, and hospice care exist between African-American and other ethnic groups.

Fossati [33]	2004	Randomized clinical trials of cytotoxic or hormonal treatments in advanced breast cancer (review of published literature before Dec 2003	QOL assessments added relatively little value to classical clinical endpoints.

Mols et al. [34]	2005	Systematic review among long-term survivors	Focusing on the long-term effects of breast cancer is important when evaluating the full extent of cancer treatment.

Grimison and Stockler [35]	2007	Adjuvant systemic therapy for early-stage breast cancer (review of literature from 1996 to Feb. 2007)	For the majority of breast cancer patients most aspects of health-related quality of life recover after adjuvant chemotherapy ends without long-term effects except vasomotor symptoms and sexual dysfunction.

Health-related quality of life in patients undergoing systemic therapy for advanced breast cancer was reviewed by Bottomley and Therasse, covering the literature from 1995 to 2001. They indicated that there were 19 studies. Among these, there were 12 studies on chemotherapy, 6 hormonal trials and 1 on biological therapy (Trastuzumab). They concluded that quality of life data provided invaluable insights into the treatment and care of patients [28].

To help the selection of optimal treatment, Goodwin et al. conducted a review of measurements of health-related quality of life in randomized clinical trials in breast cancer patients, covering the literature from 1980 to 2000. They identified a total of 256 randomized trials in breast cancer that included health-related quality of life or psychosocial outcomes. Of these, 66 trials involved randomized of different treatment options, 46 evaluated biomedical interventions and 20 evaluated psychosocial interventions. They concluded that until the results of ongoing trials are available, caution is recommended in initiating new quality of life studies unless treatment equivalence is expected or unless unique or specific issues can be addressed [30]. Similarly, Fossati's critical review of published literature on randomized clinical trials of cytotoxic or hormonal treatments of advanced breast cancer indicated that quality of life assessments added relatively little value to classical clinical endpoints [33].

Mols et al. reviewed the literature on quality of life among long-term survivors of breast cancer and found that although these patients experienced some specific problems such as a thick and painful arm and problems with sexual functioning, most reported good overall quality of life. The review also indicated that the current medical condition, amount of social support and current income level were strong positive predictors of quality of life, and the use of adjuvant chemotherapy emerged as a negative predictor. The authors concluded that focusing on the long-term effects of breast cancer is important when evaluating the full extent of treatment [34].

Grimison and Stockler reviewed quality of life in early-stage breast cancer patients receiving adjuvant systemic therapy, review of clinical randomized trials covering the literature from 1996 to 2007, and concluded that the long-term effects of chemotherapy-induced menopause and hormonal therapy on quality of life were poorly recognized. They found that vasomotor symptoms and altered sexual function were common, distressing and inadequately treated [35].

### Two historical papers

The first paper on quality of life in breast cancer patients was published in 1974. In this historical paper advanced breast cancer patients receiving adrenalectomy with chemotherapy were assessed for objective and subjective response rates, survival and quality of life. The results showed that in 64% of the patients the subjective palliation involved a return to essentially normal living during the period of improvement [36]. The second historical paper on the topic was appeared two years later, in 1976; Priestman and Baum used a linear analogue self-assessment (LASA) to measure the subjective effects of treatment in women with advanced breast cancer [37]. The results showed that this technique might be used to monitor the subjective benefit of treatment and to compare the subjective toxicities of different therapeutic regimens. The results also suggested that the subjective toxicity of cytotoxic therapy was not related to the patient's age and diminished with successive courses of drugs. However, not until the late 1980s and early 1990s was the literature gradually supplemented with papers using relatively standard and established instruments to measure quality of life in breast cancer patients.

### Instruments used

Broadly, quality of life measures can be classified as: general, disease specific, and site-specific. Although the early studies did not use standard measures, several valid instruments for measuring quality of life in breast cancer patients have been developed in recent years. The most commonly-used instruments were: the European Organization for Research and Treatment of Cancer (EORTC) Quality of Life Questionnaire and its Breast Cancer supplement (EORTC QLQ-C30 and QLQ-BR23); the Functional Assessment of Chronic Illness Therapy General Questionnaire and its Breast Cancer Supplement (FACIT-G and FACIT-B formerly FACT questionnaires); the Breast Cancer Chemotherapy Questionnaire (BCQ); the Hospital Anxiety and Depression Scale (HADS); and the Medical Outcomes Study Short Form Survey (SF-36). Table 4 lists a number of most important instruments used in studies of quality of life in breast cancer patients. Almost all these instruments proved to be valid and were found to be very popular among researchers and clinicians.

**Table 4:** A list of instruments used to measure quality of life in breast cancer patients (1974–2007)

**Types of measures**	**Measures full name**	**Abbreviation**
*General measures*		
	Short Form Health Survey	SF-36
	Spitzer Quality of Life Index	QLI
	Sickness Impact Profile	SIP
	Ferrans and Powers Quality of Life Index	QLI
*Cancer specific measures*		
	European Organization for Research and Treatment of Cancer Core quality of Life questionnaire	EORTC QLQ-C30
	Functional Assessment of Chronic Illness Therapy General Questionnaire	FACIT-G (formerly FACT)
	Functional Living Index-Cancer	FLI-C
	Ferrans and Powers Quality of Life Index-Cancer	QLI-C
*Breast cancer specific measures*		
	European Organization for Research and Treatment of Cancer Breast Cancer Quality of Life Questionnaire	EORTC QLQ-BR23
	Functional Assessment of Chronic Illness Therapy-Breast	FCIT-B
	Breast Cancer Chemotherapy Questionnaire	BCQ
	The Satisfaction with Life Domains Scale for Breast Cancer	SLDS-BC
*Psychological measures*		
	General Health Questionnaire-28	GHQ-28
	Hospital Anxiety and Depression Scale	HADS
	Beck Depression Inventory	BDI
	Center for Epidemiologic Studies Depression Scale	CES-D
	State-Trait Anxiety Inventory	STAI
	Profile Mood State	PMS
	Mental Adjustment to Cancer Scale	MACS
	Psychosocial Adjustment to Illness Scale	PAIS
*Symptom measures*		
	Functional Assessment of Chronic Illness Therapy-Fatigue	FACIT-F
	Piper Fatigue Scale	PFS
	Multidimensional Fatigue Inventory	MFI
	Functional Assessment of Chronic Illness Therapy-B plus Arm Morbidity Subscale	FACIT-B + 4
	Hot Flash Related Interference Scale	HFRDIS
	Shoulder Disability Questionnaire	SDQ
	Brief Pain Inventory	BPI
	McGill Pain Questionnaire	MPQ
	Memorial Symptom Assessment Scale	MSAS
	Rotterdam Symptom Checklist	RSC
*Other measures*		
	Functional Assessment of Chronic Illness Therapy-Spiritual	FACIT-SP
	Body Image Scale	BIS
	Body Image After Breast Cancer Questionnaire	BIBCQ
	Watts Sexual Functioning Questionnaire	WSFQ
	Social Support Questionnaire	SSQ
	Life Satisfaction Questionnaire	LSQ
	Satisfaction With Life Scale	SWLS

### Validation studies

Development of instruments for measuring quality of life in breast cancer patients, or cultural adaptation and validation studies of the existing instruments, was the major theme in a number of papers. These are presented in Table 5[38-59]. A paper by Levine et al. in 1988 was the first validation study in this field. It reported a quality of life measure in breast cancer patients called the Breast Cancer Chemotherapy Questionnaire (BCQ). This is a 30-item questionnaire that focuses on loss of attractiveness, fatigue, physical symptoms, inconvenience, emotional distress and feelings of hope and support from others [35]. A few studies reported translation and validation findings for the instruments used to assess quality of life among breast cancer patients in different cultures (for example see [48,54,56]).

**Table 5:** A summary of validation studies of quality of life instruments in breast cancer patients (1974–2007)

**Author(s) [Ref.]**	**Year**	**Instrument**	**Main focus**
Levine et al. [38]	1988	The Breast Cancer Chemotherapy Questionnaire (BCQ)	Development an outcome measure in clinical trials of adjuvant chemotherapy

Ciampi et al. [39]	1988	A 27 item Linear Analog Self Assessment	Factor analysis indicating disease and treatment-related, physical, emotional and social health summary scores

Tamburini et al. [40]	1991	Two simple index	To assess the impact of therapy on QOL in patients receiving chemotherapy for operable breast cancer

Osoba et al. [41]	1994	The European Organization for Research and Treatment of Cancer Core Quality of Life Questionnaire (EORTC QLQ-C30)	Evaluation of psychometric properties and responsiveness

Carlsson and Hamrin [42]	1996	The Life Satisfaction Questionnaire (LSQ-32)	Development a tool to measure life satisfaction in breast cancer patients

Sprangers et al. [43]	1996	The European Organization for Research and Treatment of Cancer Breast Cancer Specific Quality of Life Questionnaire (EORTC QLQ-BR23)	Development of a breast cancer specific QOL measure

Brady et al. [44]	1997	The Functional Assessment of Cancer Therapy Breast Cancer Specific Questionnaire (FACT-B)	Development of a breast cancer specific QOL measure

de Haes and Olschewski [45]	1998	The Rotterdam Symptom Checklist (RSC)	Cross cultural validation

McLachlan et al. [46]	1998	The EORTC QLQ-C30	Validation as a measure of psychological function

Fallowfiled et al [47]	1999	An endocrine symptom subscale for the FACT-B (FACT-B plus ES)	Validation in women undergoing hormonal therapy for breast cancer

Montazeri et al. [48]	2000	The EORTC QLQ-BR23	Validation of the Iranian version

Mihailova et al. [49]	2001	The EORTC QLQ-C30 and the QLQ-BR23	Validation of the Bulgarian version

Coster et al. [50]	2001	The Impact of Arm Morbidity (FACT-B+4)	Development a QOL scale to assess the impact of arm morbidity post-operatively

Carpenter [51]	2001	The Hot Flash Related Daily Interference Scale	Development of a tool for measuring the impact of hot flashes on QOL

Pandey et al. [52]	2002	The FACT Breast Cancer Specific Questionnaire (FACT-B)	Validation of the Malayalam version

Chie et al. [53]	2003	The EORTC QLQ-C30 and the EORTC QLQ-BR23	Validation of the Taiwan Chinese version

Lee et al. [54]	2004	The Functional Assessment of Cancer Therapy-General (FACT-G)	Validation of the Korean version

Yun et al. [55]	2004	The EORTC QLQ-BR23	Cross-cultural application in Korea

Parmar et al. [56]	2005	The EORTC QLQ-C30	Validation of the Indian version

Avis and Foley [57]	2006	The Quality of life in Adult Cancer Survivors (QLACS)	Evaluation in long term breast cancer survivors

Wan et al. [58]	2007	The FACT-B	Validation of the simplified Chinese version

Wan et al. [59]	2007	The EORTC QLQ-BR53	Psychometric properties of the simplified Chinese version

### Measurement issues

Papers that dealt with issues of quality of life measurement in breast cancer patients encompassed a variety of topics, mainly focusing on methodological and practical concerns in such assessment, especially in clinical settings. Most authors have tried first to convince clinicians to assess quality of life, and secondly to show how quality of life data could contribute to care and management of breast cancer patients. Table 6 presents a summary of the results [60-84].

**Table 6:** A list of quality of life studies that covered measurement issues in breast cancer patients (1974–2007)

**Author(s) [Ref.]**	**Year**	**Main focus**	**Conclusion(s)/Recommendation**
Baum et al. [60]	1990	The issue of measuring QOL in advanced breast cancer	Efforts are being made to find out ways to measure QOL in advanced breast cancer patients.

Sutherland et al. [61]	1990	Ratings of the importance of QOL variables	Breast cancer patients give different weights to different QOL variables.

Gelber et al. [62]	1992	Explaining about the QOL adjusted Time Without Symptom and Toxicity	Integration of two methods (QOL and symptom free duration) could provide a new tool.

Ganz et al. [63]	1992	The influence of multiple variables on the relationship of age to QOL	The casement plot methodology should be employed for simultaneous evaluation of multiple variables.

Gelber et al. [64]	1993	Description of survival estimates with applications to QOL evaluation (Quality adjusted Time Without Symptoms of disease and Toxicity of treatment)	Estimation showed that patients continued to benefit greatly from long-term-duration chemotherapy between 5 and 10 years following treatment.

Hyden et al. [65]	1993	Pitfalls in collecting QOL data	Several recommendations were made: (a) build support for QOL assessment among the group's leadership, (b) involve physicians and oncology nurses in the study design, (c) identify a QOL liaison at each participating institution, and (d) aggressively monitor the quality and timeliness of data submission.

Fallowfield [66]	1993	Measurement issues	Some recommendations for selecting well validated measures.

Gerard et al. [67]	1993	Framing and labeling effects in measuring quality adjusted life years	A significant difference was found in the particular values of descriptions that were written in the third person that differed in terms of whether the word "cancer" was used.

Hurny et al. [68]	1994	Timing of baseline QOL assessment	Timing is an important consideration in QOL assessment.

Fallowfield [69]	1995	Discussion on some instruments used to measure QOL	Monitoring QOL in breast cancer should be a mandatory part of follow-up in clinical trials.

Hietanen [70]	1996	Measurement and practical aspects of QOL assessment	Main factors affecting QOL in the treatment of breast cancer.

Bernhard et al. [71]	1997	The International Breast Cancer Study Group (IBCSG) approach	Confirmation of the feasibility, validity and clinical relevance of quality of life assessment.

Bernhard et al. [72]	1998	Factors affecting baseline QOL assessment	Cultural and biomedical factors are influencing baseline QOL data and should be considered when evaluating the impact of treatment.

Bernhard et al. [73]	1998	Practical issues and factors associated with missing data	The factors most highly associated with missing data were institution and chemotherapy compliance.

Ganz et al. [74]	1998	Compliance with QOL data collection	Educational level of a trial participants might contribute to it compliance.

Coates and Gebski [75]	1998	Approaches to missing data	Missing data cannot be assumed to be similar to those available. Optimal assessment requires careful prospective attention to complete data collection.

Jansen et al. [76]	2000	Response shift	Significant recalibration effects were observed.

Curran et al. [77]	2000	Summary measures and statistics	Different techniques in analysis might result in different conclusions.

Perez et al. [78]	2001	The application of a time trade-off utility measure	The utility measure and a QOL measure showed fair to moderate concordance.

Nagel et al. [79]	2001	A cluster analytic approach to analyze quality of life data	QOL scores could identify clinically meaningful subgroups of patients.

Mosconi et al. [80]	2001	A general introduction to the debate on the methodological issues involved in QOL evaluation	Open questions regarding the use of QOL measures in surgical, adjuvant therapy and metastatic studies.

Efficace et al. [81]	2002	Evaluating reliability, validity and cultural relevance of QOL measures in clinical trials	Suggestions for selecting future measures for use in breast cancer population of patients.

Wilson et al. [82]	2005	Comparing two QOL measures (the Rand 36-item and the Functional Living Index-Cancer)	Neither questionnaire can be replaced by each other in studies of QOL in breast cancer patients.

Carver et al. [83]	2006	Assessment of demographic, medical and psychological variables on outcome	Different aspects of QOL at long-term follow-up had different antecedents.

Perry et al. [84]	2007	Benefits, acceptability and utilization of QOL assessment in women with breast cancer	Summarized the benefits, challenges, and barriers of QOL measurement for female breast cancer patients.

### Surgical treatment

Breast cancer surgery including conservative surgery followed by irradiation, and modified radical mastectomy or radical mastectomy followed by immediate reconstruction is associated with different side-effects including pain, and fatigue and thus affecting quality of life in breast cancer patients. A list of studies on surgery and quality of life in breast cancer patients is given in Table 7[85-113]

**Table 7:** A list of studies of surgical treatment and quality o life in breast cancer patients (1974–2007)

**Author (s) [Ref.]**	**Year**	**Treatment (assessment time)**	**Conclusion(s)**
de Haes et al. [85]	1985	MAS vs. tumorectomy (11 months after surgery)	No differences expect worse body image in MAS patients.

de Haes et al. [86]	1986	MAS vs. tumorectomy (11 and 18 months after surgery)	Overall QOL improved over time in both groups; poor body image in MAS.

Ganz et al. [87]	1992	MAS vs. BCS after one year	No significant differences in QOL and both groups improved; BCS patients did not experience significantly better QOL but had fewer problems with clothing and body image.

Shimozuma et al. [88]	1994	Surgery-any	Hospitalization had a strong negative relation to overall QOL; type of surgery had no significant association with QOL.

Neises et al. [89]	1994	MAS or BCS	Older women suffer as much as younger patients after MAS.

Fallowfield [90]	1994	Surgery and tamoxifen vs. tamoxifen alone	At 2 years similar psychological health; no evidence of impaired QOL for elderly women after surgery

Shimozuma et al. [91]	1995	MRM or BCS (before surgery and 3 times up 2 years after)	No significant differences in overall QOL; patients with BCS need more psychological support.

Hart et al. [92]	1997	MAS + prostheses or MAS + reconstruction or MAS alone	No one technique is necessary for all women to optimize QOL; women should choose and make their own decisions.

Dorval et al. [93]	1998	Partial or total MAS (3 and 18 months after)	Both appeared to be equivalent in long-term QOL. Younger women might benefit more from partial MAS.

Curran et al. [94]	1998	MRM vs. BCS	Significant benefit in body image and satisfaction in BCS group; no difference in fear of recurrence.

Wapnir et al. [95]	1999	Lumpectomy with axillary dissection (LAD) or mastectomy	No major differences except for dressing, comfort with nudity and sexual drive in favor of ALD.

Shimozuma et al. [96]	1999	MRM or BCS (1 year after)	At one year good QOL, with no relationship to the type of surgery.

Pusic et al. [97]	1999	Lumpectomy + irradiation or MAS + reconstruction or MAS alone	Postoperative QOL varied with age; for age less than 55 QOL was lowest for MAS, over 55 was lowest for lumpectomy.

Amichetti et al. [98]	1999	BCS + irradiation in non-infiltrating breast cancer	Good QOL and body image and lack of negative impact on sexuality.

King et al. [99]	2000	MAS or BCS (3 months and 1 year after)	Most symptoms declined over time but arm and menopausal symptoms persisted; worse QOL in younger patients.

Kenny et al. [100]	2000	MAS or BCS + irradiation (1 year after)	Better body image and physical function in BCS; more impact on younger women regardless of treatment type.

Nissen et al. [101]	2001	MAS or MAS + reconstruction or BCS (6 times assessment up to 2 years after)	QOL other than body image were not better in BCS or MAS + reconstruction than in who had MAS alone; MAS + reconstruction was associated with greater mood disturbance and poorer QOL.

Janni et al. [102]	2001	MAS or BCS (median 46 months follow-up)	Surgical modalities had no long-term impact on overall QOL, but certain body image related problems in MAS was observed.

Girotto et al. [103]	2003	MAS + reconstruction in older women	Improved QOL in older patients especially improved mental health.

Cocquyt et al. [104]	2003	Skin-sparing MAS or BCS	Both yielded comparable QOL, but cosmetic outcome was better after skin-sparing MAS.

Engel et al [105]	2004	MAS or BCS (5 years follow-up)	MAS patients had lower body image, role and sexual functioning; BCS should be encouraged in all ages.

Ganz et al. [106]	2004	Lumpectomy + chemotherapy or MAS + chemotherapy or Lumpectomy alone or MAS alone in non-metastatic breast cancer patients	At the end of primary treatment all treatment groups reported good emotional functioning but decreased physical health especially among women who had MAS or received chemotherapy.

Dubernard et al. [107]	2004	SLNB	Axillary procedure affected only QOL related to arm morbidity.

Elder et al. [108]	2005	MAS + immediate breast reconstruction (before and 12 months after)	After 12 months good QOL comparable with aged-matched women from the general population.

Barranger et al. [109]	2005	SLNB vs. ALND in breast-sparing treatment	SLNB was associated with significantly lower mid term morbidity.

Fleissig [110]	2006	SLNB vs. ALND	Regarding arm functioning and QOL the use of SNB was recommended in patients with node negative breast cancer.

Pandey et al. [111]	2006	MAS or BCS	No significant change in overall QOL after surgery; poorer QOL in MAS patients.

Rietman et al. [112]	2006	SLNB or ALND (before and after 2 years)	Less treatment related upper limb morbidity, perceived disability in activities of daily life and worsening of QOL after SNLB compared with ALND.

Parker et al. [113]	2007	MAS or MAS+ reconstruction or BCS (short- and long-term effects on aspects of psychosocial adjustment and QOL	Overall, the general patterns of psychosocial adjustment and QOL were similar among the three surgery groups.

Abbreviations

MRM: modified radical mastectomy, MAS: mastectomy, BCS: breast conservation surgery, SNLB: sentinel lymph node biopsy, ALND: axillary lymph node dissection

The most important topic in studies of breast cancer surgery and quality of life relates to the type of surgery. Recent findings suggest that partial and total mastectomy appear to be equivalent treatments in terms of patients' long-term quality of life. However, both short-term and long-term distress levels after partial and total mastectomy may depend on patient's age at diagnosis [93]. A study of early breast cancer patients one year after mastectomy or conservative surgery and radiation therapy found that the differences between treatment groups were mainly accounted for by adjuvant therapies. Those treated by breast conservation reported better body image but worse physical functions. The negative impact of breast cancer and its treatment was greater for younger women across a number of dimensions of quality of life measures regardless of treatment type [100].

In addition, one study found that aspects of quality of life other than body image were no better in women who underwent breast-conserving surgery or mastectomy with reconstruction than in women who had mastectomy alone. Furthermore, mastectomy with reconstruction was associated with greater mood disturbance and poorer health [101]. However, the results of a 5-year prospective study on quality of life following breast-conserving surgery or mastectomy indicated that mastectomy patients had a significantly worse body image; role and sexual functioning, and their lives were more disrupted [105]. A recent Japanese study on the early effects of surgery in patients with breast cancer performing multivariate analysis reported that there were no significant differences in quality of life before and after surgery, but quality of life was significantly better among women undergoing breast conservation than those undergoing mastectomy [111]. A study comparing the short- and long-term effects of mastectomy with reconstruction, mastectomy without reconstruction, and breast conservation therapy on aspects of psychosocial adjustment and quality of life in a sample of 258 women with breast cancer concluded that overall, the general patterns of psychosocial adjustment and quality of life were similar among the three surgery groups. In addition the study results showed that during the long-term follow-up period (6 months to 2 years after surgery), women in all three groups experienced marked improvements in psychosocial adjustment (depressive symptoms, satisfaction with chest appearance, sexual functioning) and quality of life in physical and mental health domains [113].

### Systemic therapies

In order to reduce the risk of recurrence and death, breast cancer patients usually receive systemic therapies (chemotherapy, hormonal therapy and biological treatments) after surgery. Several studies evaluated quality of life in breast cancer patients receiving systemic therapies. A list of studies reporting on the topic is given in Table 8[36,37,114-169].

**Table 8:** A list of studies on systemic therapies and quality of life in breast cancer patients (1974–2007)

**Author(s) [Ref.]**	**Year**	**Treatment/patients**	**Conclusion(s)**
Moore et al. [36]	1974	Adrenalectomy + chemotherapy in advanced breast cancer	In most patients the subjective palliation involved a return to normal living.

Priestman and Baum [37]	1976	Chemotherapy in advanced breast cancer	Toxicity is not related to the patients' age and diminished with successive courses of drugs.

Palmer et al. [114]	1980	A single agent vs. five drug combination in node positive primary breast cancer	Better QOL in single agent group.

Coates et al. [115]	1987	Intermittent vs. continuous chemotherapy in metastatic breast cancer	Continuous chemotherapy was better; changes in the QOL were independent prognostic factor of survival.

Kiebert et al. [116]	1990	Peri-operative chemotherapy vs. no chemotherapy in early stage breast cancer	No differences 1 year after; patients considered chemotherapy most burdensome aspect of treatment.

Gelber et al. [117]	1991	Single cycle of combination chemotherapy vs. longer duration chemotherapy for pre-menopausal or chemo-endocrine therapy for postmenopausal women	Better QOL in longer duration chemotherapy or chemo-endocrine therapy.

Berglund et al. [118]	1991	Late effects of adjuvant chemotherapy vs. postoperative radiotherapy in pre- and post-menopausal breast cancer	Chemotherapy patients had higher overall QOL.

Richards et al. [119]	1992	A (weekly for 12 courses vs. every three weeks for 4 courses) in advanced breast cancer	Similar survival but higher psychological distress in the three weeks group.

Hurny et al. [120]	1992	CMF (6 cycles vs. 3 cycles) in operable breast cancer	QOL improved with increasing time from the study entry.

Campora et al. [121]	1992	Adjuvant chemotherapy vs. palliative chemotherapy in metastatic breast cancer	No significant difference between groups.

Fraser et al. [122]	1993	CMF vs. E in advanced breast cancer	Similar survival and no significant difference in overall global QOL.

Twelves et al. [123]	1994	Iododoxorubicin in advanced breast cancer	Little evidence of benefit in terms of physical symptom relief, level of activity, psychological symptoms or global QOL.

Bertsch and Donaldson. [124]	1995	Vinorelbine vs. melphalan	Vinorelbine was better in some aspects of QOL.

Swain et al. [125]	1996	AC + G-CSF in node positive breast cancer	Tolerable physical symptoms and emotional distress.

McQuellon et al. [126]	1996	High-dose chemotherapy + ABMT	No significant difference between pre- and post-treatment QOL.

Larsen et al. [127]	1996	High-dose chemotherapy + ASCT	Resulting in poor physical and emotional health.

Hurny et al. [128]	1996	6 cycles of CMF vs. 3 cycles CMF in node-positive operable breast cancer	Worse QOL during treatment but not after treatment completion.

Griffiths and Beaver [129]	1997	High-dose chemotherapy in advanced breast cancer	No significant deterioration in QOL.

Lindley et al. [130]	1998	Systemic adjuvant therapy	2–5 years after treatment good QOL. Small to modest gain was acceptable to women.

Ganz et al. [131]	1998	TAM or chemotherapy alone or chemotherapy + TAM, or no adjuvant therapy	No significant differences in global QOL among treatment groups; those who received chemotherapy had more sexual problems and those who received TAM had more vasomotor symptoms.

Bernhard et al. [132]	1999	Formestane vs. megestrol acetate in postmenopausal advanced breast cancer while on TAM	No significant difference in QOL; baseline QOL was strong predictive for QOL under treatment but not for time to treatment failure.

Fairclough et al. [133]	1999	CAF vs. dose intensive a 16-week multi-drug regimen	Negative impact of the dose intensive 16-week regimen was observed, although Q-TwiST analysis showed a small gain for this regimen.

Osoba and Burchmore [134]	1999	Trastuzumab (Hercptin) in metastatic breast cancer who may or may not have had prior chemotherapy	Trastuzumab was associated with an amelioration of the deleterious effects of chemotherapy alone; the drug was not associated with worsening of QOL.

McLachlan et al. [135]	1999	Chemotherapy in metastatic breast cancer	QOL maintained or improved; patients did not want to trade quantity for QOL.

Macquart-Moulin et al. [136]	2000	High-dose chemotherapy + G-CSF + ASCT in inflammatory breast cancer	QOL deterioration disappeared after treatment and returned to baseline after one year.

Riccardi et al. [137]	2000	Doubling E within FEC vs. FEC in metastatic breast cancer	No significant difference in response or improvement of baseline QOL.

Kramer et al. [138,139]	2000	Paclitaxel vs. A in advanced breast cancer	QOL appeared to be prognostic for survival and response to treatment.

Joly et al. [140]	2000	CMF + irradiation vs. irradiation in pre-menopausal breast cancer	Similar QOL was observed.

Hakamies-Blomqvist et al. [141]	2000	T vs. sequential MF in metastatic breast cancer	Difference in QOL was minor favoring MF.

Broeckel et al. [142]	2000	Adjuvant chemotherapy treated breast cancer (after 3 to 36 months)	Younger age, unmarried status, time since diagnosis and chemotherapy completion related to greeter depressive symptoms.

Carlson et al. [143]	2001	High-dose chemotherapy + ASCT in metastatic breast cancer	Anxiety and depression continued to increase, loss of sexual interest, worrying and joint pain were reported.

Osoba et al. [144]	2002	Chemotherapy + Trastuzumab (Hercptin) vs. Chemotherapy alone in metastatic breast cancer	More improved global QOL with chemotherapy + Herceptin.

Modi et al. [145]	2002	Paclitaxel in metastatic breast cancer	QOL benefit in tumor response patients.

Heidemann et al [146].	2002	Mitoxantrone vs. FEC in metastatic breast cancer	No significant difference in survival or response but a QOL scores favored mitoxantrone.

Genre et al. [147]	2002	High-dose-intensity AC (21 vs. 14 days)	Shortening cycles had a high negative impact on QOL.

de Haes et al. [148]	2003	Goserelin vs. CMF in peri-and pre-menopausal node-positive early breast cancer	Better QOL in favor of goserelin.

Brandberg et al. [149]	2003	Tailored FEC vs. induction FEC followed with high-dose CTCb + peripheral SCT	No significant overall differences were found between groups.

Land et al. [150]	2004	CMF vs. AC in axillary node negative and estrogen receptor negative breast cancer	Overall QOL was equivalent between two groups.

Fallowfield et al. [151]	2004	ANA vs. TAM alone or in combination in postmenopausal early breast cancer	Similar overall QOL impact but some small differences in side effects profiles.

Bottomely et al. [152]	2004	AT vs. AC in metastatic breast cancer	No significant differences in QOL between two groups.

Bernhard et al. [153]	2004	TAM for 5 years or three prior cycles of CMF followed by 57 months TAM in estrogen receptor-negative and estrogen receptor-positive breast cancer	At completion there were no differences by treatment groups.

Tong et al. [154]	2005	Capecitabine, idarubicin and cyclophosphamide (all-oral regimen, XIC) in metastatic breast cancer	No significant decease in global QOL scores.

Galalae et al. [155]	2005	Radiotherapy and adjuvant chemotherapy vs. radiotherapy and hormonal therapy vs. radiotherapy alone after conserving surgery	Adjuvant chemotherapy lowered QOL vs. hormones or radiotherapy alone.

Elkin et al. [156]	2005	Ovarian suppression vs. chemotherapy in pre-menopausal hormone-responsive breast cancer	Assuming equal efficacy ovarian suppression was superior. Efficacy would have impact on treatment choice.

Conner-Spady et al. [157]	2005	High-dose chemotherapy + ABST in breast cancer with poor prognosis	Impaired QOL in short term but improved after 2 years.

Bottomley et al. [158]	2005	Dose-intensives chemotherapy (CE + filgrastim) vs. CEF in locally advanced breast cancer	Groups did not differ in progression free survival; lower QOL in intensified group at short term but no difference at long term.

Ahles et al. [159]	2005	Standard-dose systemic chemotherapy vs. local therapy only in long-term breast cancer survivors	Lower overall QOL in chemotherapy group.

Peppercorn et al. [160]	2005	High-dose chemotherapy + ABMT vs. intermediate-dose chemotherapy in patients with stage II and III breast cancer	Patients who received more intensive therapy experienced transient declines in QOL; by 12 months after, QOL was comparable between the 2 arms, regardless of therapy intensity, and many QOL areas were improved from baseline.

Semiglazov et al. [161]	2006	CMF + mistletoe lectin (PS76A2) vs. CMF + placebo	PS76A2 improved QOL during and after chemotherapy.

Martin et al. [162]	2006	FAC vs. TAC or TAC + G-CSF in node negative breast cancer	Lower QOL in patients treated with TAC. Addition of G-CSF improves QOL.

Hurria et al. [163]	2006	Anthracyclin-based chemotherapy or CMF in older women with breast cancer	QOL maintained in both group.

Fallowfield et al. [164]	2006	EXE vs. TAM after 2–3 years of TAM in postmenopausal primary breast cancer	Temporary decrease in overall QOL for EXE but no other differences.

Groenvold et al. [165]	2006	CMF vs. ovarian ablation	CMF had more negative impact on QOL.

Cella et al. [166]	2006	ANA vs. TAM alone or in combination in postmenopausal breast cancer	ANA and TAM had similar impact on QOL.

Liu et al. [167]	2006	DPPE + A vs. A in patients with advanced or metastatic breast cancer	Patients on A alone had fewer disease and treatment adverse events and better QOL.

Karamouzis et al. [168]	2007	Chemotherapy vs. supportive care in metastatic patients	QOL was better in patients receiving chemotherapy than those under supportive care.

Hopwood et al. [169]	2007	Adjuvant radiotherapy	QOL and mental health were favorable for most patients about to start radiotherapy but younger age and receiving chemotherapy were significant risk factors for poorer QOL.

Abbreviations

C: Cyclophosphamide, M: Methotrexate, F: 5-fluorouracil, A: Doxorubcin, E: Epirubcin, T: Docetaxel, TAM: Tamoxifen, ANA: Anastrozole, EXE: Exemestane, QOL: Quality of life, DPPE: Tesmilifene, Granulocyte colony stimulating factor: G-CSF, CTCb: Cyclophosphamide, thiotepa, and carboplatin

Chemotherapy has considerable effect on quality of life of breast cancer patients. In a study of postoperative adjuvant chemotherapy in primary node positive breast cancer patients (one or more axillary node), women receiving a single agent or a multi-drug regimen indicated that the treatment was *'unbearable' *[114] or in a study of patients with early breast cancer receiving preoperative chemotherapy almost all patients considered chemotherapy the most *'burdensome' *aspect of the treatment [116].

The side-effects of chemotherapy on quality of life in breast cancer patients were the topic of many investigations. In these studies, investigators looked at the issue from different perspectives. For instance, using a decision-analytic approach to evaluate tradeoffs between efficacy and quality of life in the choice of three adjuvant treatments (chemotherapy, surgical ovarian suppression, and medical ovarian suppression) in pre-menopausal women with newly-diagnosed, hormone-responsive early breast cancer, Elkin et al. concluded that when different treatments have similar efficacy, there may be a subgroup of women for whom quality of life considerations dominate the choice. However, they stated that small differences in the relative efficacy of these therapies have a substantial impact on treatment choice [156].

To improve clinical outcomes an international randomized controlled trial compared dose-intensive chemotherapy with standard systemic chemotherapy in patients with locally advanced breast cancer and showed that a dose-intensive regimen only has a temporary effect on health-related quality of life, thus enabling more research on intensive treatment for patients with locally advanced breast cancer, as it might also offer a survival benefit [158].

However, recent studies focusing on adjuvant hormonal therapies (tamoxifen or aromatase inhibitors such as anastrozole, letrozole, exemestane) and quality of life in postmenopausal early-stage breast cancer patients reported more encouraging results. Most studies found that overall quality of life was improved in patients receiving either anstrozole or tamoxifen but patients reported different side effects [151,166]. A trial comparing tamoxifen with exemestane showed that quality of life did not change significantly in either groups, but there were improvements in endocrine-related symptoms [164].

In summary, as noted by Grimison and Stockler, for the majority of breast cancer patients most aspects of health-related quality of life recover after adjuvant chemotherapy ends without long-term effects except vasomotor symptoms and sexual dysfunction. However, tamoxifen and aromatase inhibitors cause long-term effects due to vasomotor, gynecological and sexual problems [35].

### Quality of life as predictor of survival

Until recently, only a few studies had reported a relationship between quality of life and survival in breast cancer patients [115]. A study using the Daily Diary Card to measure quality of life in advanced breast cancer showed that the instrument offered accurate prognostic data regarding subsequent response to treatment and survival duration [170]. Similarly, Seidman et al. evaluated quality of life in two phase II clinical trials of metastatic breast cancer and found that baseline scores of two validated quality of life instruments independently predicted the overall likelihood of tumour responses [171].

Studies have shown that baseline quality of life predicts survival in advanced breast cancer but not in early stage of disease [172]. Two recently published papers also confirmed that baseline quality of life is not a prognostic factor in non-metastatic breast cancer patients. One of these two studies, using Cox survival analysis, indicated that neither health-related quality of life nor psychological status at diagnosis or 1 year later was associated with medical outcome in women with early-stage breast cancer [173]. The other study with a sample of 448 locally advanced breast cancer patients, reported that baseline health-related quality of life parameters had no prognostic value in a non-metastatic breast cancer population [174]. However, other studies have demonstrated that some aspects of quality of life data including physical health [175], pain [139,176], and loss of appetite [177] were significant prognostic factors for survival in women with advanced breast cancer. In addition, one study demonstrated that baseline physical aspects of quality of life and its changes were related to survival, but psychological and social aspects were not [178].

### Psychological distress

Women with breast cancer might develop psychological distress including anxiety and depression during diagnosis and treatment and after treatment. The psychological impact of breast cancer has received considerable attention. Since this is a separate topic, the focus here is on psychological distress as it relates to quality of life studies in breast cancer patients. Table 9 summarizes the papers on the topic [179-210].

**Table 9:** A list of studies on psychological distress and quality of life in breast cancer patients (1974–2007)

**Author (s) [Ref.]**	**Years**	**Main focus**	**Results/conclusion(s)**
Ferrero et al. [179]	1994	Mental adjustment to cancer in newly-diagnosed non-mtastatic breast cancer(an xploratory study)	Strong association between mental adjustment to cancer and reported vague physical symptoms; fighting spirit and denial was associated with better QOL and helpless/hopeless and anxious preoccupation and fatalism were negatively correlated with well-being.

Ganz et al. [180]	1996	Psychosocial concerns 2 and 3 years after primary treatment	Problems associated with physical and recreational activities, body image, and sexual functions were observed, although many positive aspects from cancer experience were reported.

Maunsell et al. [181]	1996	Brief psychological intervention vs. Brief psychological intervention + psychological distress screening	Distress screening did not improve QOL. Minimal psychological intervention at initial treatment alone was recommended.

Andrykowski et al. [182]	1996	Psychological adjustment in women with breast cancer or benign breast problems	Breast cancer patients reported poorer physical health but greater positive psychosocial adaptation and improved life outlook, no difference in psychological distress between two groups.

Marchioro et al. [183]	1996	Evaluation of the impact of a psychological intervention vs. standard care in non-metastatic breast cancer patients	Cognitive psychotherapy and family counseling improved both depression and QOL indexes.

Weitzner et al. [184]	1997	QOL and mood in long-term breast cancer survivors	Psychological measures were found to be more robust predictors of QOL than the demographic variables; long-term survivors continue to experience significant depression and lower QOL.

Kissane et al. [185]	1998	Psychological morbidity in early-stage breast cancer	45% (135/303) had psychiatric disorder, 42% had depression, anxiety or both; QOL was substantially affected.

Bloom et al. [186]	1998	Intrusiveness of illness in young women with newly-diagnosed breast cancer	Intrusiveness of illness mediated the effect of disease and treatment factors on QOL; neither time post-diagnosis nor type of treatment affected the psychological component of QOL.

Longman et al. [187]	1999	Psychological adjustment over time	Over time depression burden and anxiety burden persist and each was negatively associated with overall and present QOL.

Cotton et al. [188]	1999	Relationship among spiritual well-being, QOL, and psychological adjustment	Spiritual well-being was correlated with both QOL and psychological adjustment, but relationship was found to be more complex and indirect than previously considered.

Ashing-Giwa [189]	1999	Psychological outcome in long-term survivors of breast cancer (focus on African-American)	Patients relied on spiritual faith and family support to cope; socio-cultural contexts of the women's lives need to be considered when studying QOL.

Lewis et al. [190]	2001	Cancer-related intrusive thoughts and social support	In women with social support cancer-related intrusive thoughts had no significant negative impact on QOL, but in women with low social support there was negative effect on QOL.

Amir and Ramati [191]	2002	Post-traumatic distress disorder (PTSD), QOL, and emotional distress in long term survivors of breast cancer and a control group	Higher PSTD, emotional distress and lower QOL in breast cancer mainly due to chemotherapy and disease stage.

Ganz et al. [192]	2003	Psychosocial adjustment 15 months after diagnosis in older women with breast cancer	Psychosocial adjustment at 15 months was predicted by better mental health, emotional social support and better self-rated interaction with health care providers.

Bordeleau et al. [193]	2003	Randomized trial of group psychological support vs. control in metastatic breast cancer	Supportive-expressive group therapy did not appear to influence QOL.

Badger et al. [194]	2004	Depression burden and psychological adjustment	Depression burden had negative effect on psychological adjustment and QOL.

Schreier and Williams [195]	2004	Anxiety in women receiving either radiation or chemotherapy for breast cancer	No significant differences for total QOL or any subscales by treatment; trait anxiety was higher for chemotherapy patients; state anxiety was high and did not decrease over the course of the treatment for either group.

Kershaw et al. [196]	2004	Coping strategies in advanced breast cancer patients and their family caregivers	Patients use more emotional support, religion and positive reframing strategies while family use more alcohol or drug. In both active coping was associated with higher QOL.

Lehto et al. [197]	2005	Psychological stress factors as predictors of QOL in patients receiving surgery alone vs. adjuvant treatment	Psychosocial factors were strongest predictors of QOL but not cancer type or treatment; non-cancer related stresses showed strongest QOL decreasing influence.

Roth et al. [198]	2005	Affective distress in women seeking immediate vs. delayed breast reconstruction after mastectomy	Women seeking immediate breast reconstruction showed relatively higher psychological impairment and physical disability.

Okamura et al. [199]	2005	Psychiatric disorders and associated factors after first breast cancer recurrence	Patients' psychiatric disorders were associated with lower QOL.

Golden-Kreutz et al. [200]	2005	Traumatic stress, perceived global stress, and life events	Initial stress at diagnosis predicted both psychological and physical health at follow-up.

Deshields et al. [201]	2005	Emotional adjustment (at 4 points in time)	Primary psychological changes occur quickly after treatment conclusion and then it appeared to become stabled.

Laid law et al. [202]	2005	Self-hypnosis or Japanese healing or. control	Positive change in anxiety level, a general increase in mood and a better QOL were observed.

Schou et al. [203]	2005	Dispositional optimism and QOL.	Optimism was predictive for better emotional and social functioning one year after surgery; at time of diagnosis and throughout post-diagnosis dispositional optimism was associated with better QOL and fewer symptoms.

Grabsch et al. [204]	2006	Psychological morbidity in advanced breast cancer	42% (97/277) had a psychiatric disorder, 36% depression or anxiety or both. QOL was substantially affected.

Antoni et al. [205]	2006	Stress management after treatment for breast cancer	Stress management skill taught had beneficial effects on reduced social disruption, and increased emotional well-being, positive states of mind, benefit finding, positive lifestyle change, and positive affect.

Wonghongkul et al. [206]	2006	Uncertainty appraisal coping	Social support was used most to cope and confront-coping used the least; year of survival, uncertainty in illness and harm appraisal influenced QOL.

Yen et al. [207]	2006	Depression and stress in breast cancer versus benign tumor	Stress from health problem was the most significant predictor for QOL among malignant group.

Costanzo et al. [208]	2007	Adjustment to life after treatment	While breast cancer survivors demonstrated good adjustment on general distress following treatment, some women were at risk for sustained distress.

Wong and Fielding [209]	2007	Change in psychological distress and change in QOL	The magnitude of change in psychological distress significantly impacted physical and functional, but not social QOL in breast cancer patients.

Meneses et al. [210]	2007	Psycho-educational intervention and QOL	Breast cancer education intervention is an effective intervention in improving QOL during the first year of breast cancer survivorship.

Psychological distress in breast cancer patients is mostly related to depression, anxiety, and low emotional functioning and almost all studies have shown that psychological distress contributed to impaired quality of life especially emotional functioning, social functioning, mental health and overall quality of life. The diagnosis of the disease, importance of fears and concerns regarding death and disease recurrence, impairment of body image, and alteration of femininity, sexuality and attractiveness are factors that can cause unexpected psychological distress even years after diagnosis and treatment [211-213].

Studies have shown that psychological factors predict subsequent quality of life [200] or even overall survival in breast cancer patients [214]. A study showed that patients with lower coping capacity reported higher prevalence of symptoms, experienced higher levels of distress, and experienced worse perceived health, which in turn decreased their quality of life [215]. Furthermore, it has been shown that psychological adjustment such as the ability to cope with the disease, treatment and effects of treatment could improve outcome. The relationship between positive thinking and longer survival and a better quality of life is well documented [216].

### Supportive care

A variety of topics were covered to address supportive care issues in breast cancer patients. These ranged from papers on controlling emesis to papers that reported issues related to counseling, social support and exercise to improve quality of life [217-253]. The results are summarized in Table 10.

**Table 10:** A list of quality of life studies covering supportive care topics in breast cancer patients (1974–2007)

**Author (s) [Ref.]**	**Year**	**Intervention**	**Results/conclusion(s)**
van Holten-Verzantvoort et al. [217]	1991	Pamidronate vs. control to reduce skeletal morbidity	Less short-term mobility impairment and bone pain in treatment group but not at long term.

Young-McCaughan and Sexton [218]	1991	Aerobic exercise	Higher QOL in women who exercised.

Soukop et al. [219]	1992	Ondansetron vs. metoclopramide to control emesis	Ondansetron was significantly superior.

Kornblith et al. [220]	1993	Megestrol acetate in dose-response trial to prevent appetite loss	Lower dose was optimal achieving fewest side effects and a better QOL.

Clavel et al. [221]	1993	Ondansetron to control emesis (review of five randomized trials)	Ondansetron provided significant QOL benefits compared with metoclopramide and alizapride)

Ashbury et al. [222]	1998	One-on-one peer support (Reach to Recovery programme)	Patients were satisfied and the programme had incremental benefits to QOL of patients.

Lee [223]	1997	Social support (Reach to Recovery programme)	Social support plays a vital role in promoting overall QOL.

Wengstrom et al. [224]	1999	Nursing intervention vs. control	No measurable effect on side effects or QOL but proved to have a positive effect in minimizing stress.

Lachaine et al. [225]	1999	Ondansetron or metoclopramide to control emesis	Emesis control was significantly better in ondansetron; global QOL decreased more with metoclopramide.

Ritz et al. [226]	2000	Advanced nursing care (APN)+ standard care vs. standard care	APN improved some QOL indicators.

Molenaar et al. [227]	2001	Decision support to help patients to choose mastectomy or breast conservation	Decision-making improved as evaluated in terms of satisfaction and QOL.

Sammarco [228]	2001	Perceived social support and uncertainty in younger breast cancer survivors	Significant positive correlation between perceived social support and QOL, and significant negative correlation between uncertainty, and QOL.

Michael et al. [229]	2002	Social networks	Pre-diagnosis level of social integration was important factor in future QOL, and explains more of the variance than treatment or tumour characteristics.

Olsson et al. [230]	2002	Erythropoietin (randomized to two different doses epoetin-beta) for treatment of anemia	Global QOL was significantly improved and there was no difference between two study arms.

O'Shaughnessy [231]	2002	Effects of epoetin-alfa to prevent neuronal apoptosis vs. placebo	Improved cognitive function, mood and QOL in treatment group.

Graves et al. [232]	2003	8-week intervention based on social cognitive theory vs. standard care	Women in intervention group improved more on QOL, mood, self-efficacy, and outcome expectations.

Courneya et al. [233]	2003	Exercise training (randomized trial)	Exercise training had beneficial effects on QOL.

Turner [234]	2004	Seated exercise	Reduced fatigue and improved QOL observed.

Headley et al. [235]	2004	Effect of seated exercise vs. control	Women with advanced breast cancer randomized to the seated exercise had a slower decline in total physical well-being and less increase in fatigue.

Weinfurt et al. [236]	2004	Zoledronic asid or pamidornate disodium for metastatic bone lesion	Overall increase in QOL was observed.

Diel et al. [237]	2004	Ibandronate vs. placebo in breast cancer with metastatic bone pain	A significant improvement in QOL was observed in intervention group; fatigue and pain were also reduced.

Body et al. [238]	2004	Ibandronate vs. placebo in breast cancer with metastatic bone pain	Oral ibandronate had beneficial effects on bone pain and QOL and was well tolerated.

Wardley et al. [239]	2005	Zoledronic acid in community setting vs. hospital setting in breast cancer patients with bone metastases	No difference between settings; safety and QOL benefits were observed.

Yoo et al. [240]	2005	Muscle relaxation training and guided imagery vs. control	Less anticipatory and post-chemotherapy nausea and vomiting and higher QOL in intervention group.

Manning-Walsh [241]	2005	Relationships between persona land religious support and symptom distress and QOL	Personal support was positively related to QOL and had partial mediated effects on symptom distress but religious support was not.

Gordon et al. [242]	2005	Home-based physiotherapy or group-based exercise or no intervention	Physiotherapy was found beneficial for functioning, physical and overall QOL.

Kendall et al. [243]	2005	Influence of exercise (13.2 years following diagnosis)	High level of functioning was observed; those whose exercise increased, maintained a better QOL.

Chang et al. [244]	2005	Effect of weekly epoetin alfa on maintaining hemoglobin levels, and reduction of transfusion vs. standard care	Epoetin alfa improved QOL, maintained hemoglobin levels and reduced of transfusion.

Hudis et al [245]	2005	Effect of weekly epoetin alfa on hemoglobin levels	Epoetin alfa improved hemoglobin levels, and QOL in mildly anemic patients.

Badger et al. [246]	2005	Telephone interpersonal counseling (TPC) vs. usual care	TIP-C was partially effective in symptom management and improved QOL.

Cheema and Gual [247]	2006	Full-body exercise training (before and after evaluation study)	Significant improvements were observed in upper- and lower-body strength, endurance, and QOL.

Sutton and Erlen [248]	2006	Mutual dyadic support intervention	Most dyadic relationships were supportive, some reciprocal and some experienced conflicts.

Round et al. [249]	2006	Recovery advice to prevent treatment problems	Recovery advice given to women neither was supported nor refuted to be able improve QOL.

Giese-Davis et al. [250]	2006	Peer counseling intervention (newly diagnosed and peer counselors)	Significant improvement in newly diagnosed was observed in trauma symptoms, emotional well-being, and self-efficacy but increased emotional suppression and declined QOL in peer counselors.

Moadel et al. [251]	2007	Effects of yoga on QOL	Yoga was associated with beneficial effects on social functioning among breast cancer survivors.

Hartmann et al. [252]	2007	Effects of a step-by-step inpatient rehabilitation programme and QOL	Although not generally superior to conventional inpatient rehabilitation programmes, the step-by-step rehabilitation provided marked benefits for patients with cognitive impairments.

Kim et al. [253]	2007	Effect of complex decongestive therapy (CDT) on edema and QOL in breast cancer patients with unilateral leymphedema	CDT for upper limb lymphedema resulted in significant improved edema and QOL.

### Symptoms

There were studies on breast cancer symptoms and their relationship to quality of life. Most of these studies were related to fatigue, lymphedema, pain, and menopausal symptoms. The results are summarized in Table 11[254-280].

**Table 11:** A list of studies of quality of life and common symptoms in breast cancer patients (1974–2007)

**Author (s) [Ref.]**	**Year**	**Main focus**	**Results/conclusion(s)**
Hann et al. [254]	1998	Fatigue following radiotherapy	Women experienced fatigue but not worse than expected.

Carpenter et al. [255]	1998	Hot flushes	65% (n = 114) reported ht flushes, with 59% of women with hot flushes rating the symptom as severe; hot flushes were most severe in women with a higher body mass index, those who were younger at diagnosis, and those receiving tamoxifen.

Hann et al. [256]	1999	Fatigue after high-dose therapy and autolougous stem cell rescue	Fatigue was related to medical and psychosocial factors.

Velanovich and Szymanski [257]	1999	Lymphedema	Lymphedema occurred in a minority of patients and negatively affected QOL.

Bower et al. [258]	2000	Fatigue, occurrence, and correlates	About one-third (n = 1957) reported more severe fatigue which was associate with higher level of depression, pain, and sleep difficulties.

Kuehn [259]	2000	Surgery related symptoms following ALND	Shoulder-arm morbidity following ALND was found to be the most important long-term sources of distress.

Stein et al. [260]	2000	Hot flushes	Hot flushes have a negative impact on QOL that may be due to fatigue and interference with sleep.

Beaulac et al. [261]	2002	Lymphedema in survivors of early-stage breast cancer	MAS or BCS patients had similar lymphedema rates (28%–42/151) and had negative impact on long-term QOL in survivors.

Kwan et al. [262]	2002	Arm morbidity after curative breast cancer treatment	Symptomatic patients and patients with lymphedema had impaired QOL compared to patients with no symptoms.

Fortner et al. [263]	2002	Sleep difficulties	Most patients had significant sleep problems that frequently being disturbed by pain, nocturia, feeling too hot, and coughing or snoring loudly; patients having significant sleep problems had greater deficits in QOL.

Engel et al. [264]	2003	Arm morbidity	Up to 5 years after diagnosis 38% (n = 990) were still experienced arm problems and for these patients QOL was significantly lower than patients without arm morbidity; extent of axilla, younger age, and operating clinic significantly contributed to arm morbidity.

Caffo et al. [265]	2003	Pain after surgery	Pain distressed 40% of patients (n = 529) regardless of treatment type and had negative effect on patients' QOL.

Rietman et al. [266]	2004	Impairments and disabilities (2.7 years after surgery)	Pain was the most frequent assessed impairment after breast cancer treatment with strong relationship to perceived disability and QOL.

Schults et al. [267]	2005	Menopausal symptoms	Menopausal signs and symptoms may not be different or the breast cancer survivors and they should not be confused with the QOL/psychosocial issues of the cancer survivors.

Ridner [268]	2005	Lymphedema	Survivors with lymphedema reported poorer QOL; a symptom cluster including limb sensation, loss of confidence in body, decreased physical activity, fatigue and psychological distress was identified.

Conde et al. [269]	2005	Menopausal symptoms	Prevalence of menopausal symptoms was similar in women with and without breast cancer; sexual activity was less frequent in breast cancer patients.

Burckhardt et al. [270]	2005	Pain	Widespread pain significantly caused more experience of pain severity, pain impact and lower physical health than regional pain.

Mills et al. [271]	2005	Fatigue	Pre-chemotherapy and chemotherapy induced inflammation were related to fatigue and QOL.

Massacesi [272]	2006	Effects of endocrine related symptoms in breast cancer who had switched from tamoxifen to anastrozole	Endocrine related symptoms improved but higher rate of mild arthritic and bone pain were reported.

Land et al. [273]	2006	Tamoxifen or raloxifene related symptoms	No significant differences between groups; tamoxifen group reported better sexual function, more gynecological problems and vasomotor symptoms while raloxifene group reported more musculoskeletal problems and weight gain.

Heidrich et al. [274]	2006	Symptoms, and symptom beliefs in older breast cancer patients vs. older women without breast cancer	Symptom experience and QOL of older breast cancer survivors were similar to those of older women with other chronic health problems.

Gupta et al. [275]	2006	Menopausal symptoms	96% reported vasomotor, 83% psychological and 90% somatic symptoms (n = 200) which negatively correlated not only their own but also with their partners' QOL.

Byar et al. [276]	2006	Fatigue	Fatigue was associated with other physical and psychological symptoms and higher fatigue compromised QOL.

Arndt et al. [277]	2006	Fatigue	Fatigue emerged as the strongest predictor of QOL.

Pyszel et al. [278]	2006	Disability, and psychological distress in breast cancer survivors with and without lymphedema	Patients with arm lymphedema were more disabled, experienced a poorer QOL and had increased psychological distress in comparison to those without lymphedema.

Dagnelie et al. [279]	2007	Fatigue	Of all QOL domains/subscales, fatigue is by far the predominant contributor to patient-perceived overall QOL in breast cancer patients preceding high-dose radiotherapy.

Janz et al. [280]	2007	Relationship between symptoms and post-treatment QOL	Five most common symptoms were: systemic therapy side effects, fatigue, breast symptoms, sleep difficulties, and arm symptoms. Fatigue had the greatest impact on QOL.

ALND: axillary lymph node dissection, ASCT: autologous stem cell transplantation, SLNB: sentinel lymph node biopsy.

Fatigue is the least definable symptom experienced by patients with breast cancer and its effect on impaired quality of life cannot be explained precisely. A recent publication studying 1,588 breast cancer patients showed that fatigue (as measured by the EORTC QLQ-C30 fatigue subscale) independently predicted longer recurrence-free survival when biological factors were controlled in the analysis. When combined with the biological model, fatigue still remained a significant predictor of recurrence-free survival [214].

### Sexual functioning

Breast cancer could be regarded as a disease that relates to women's identities. In this respect, sexual functioning is an important issue, especially in younger breast cancer patients. Among quality of life studies in breast cancer patients only six papers focused especially on sexual functioning [281-286]. The findings indicated that disrupted sexual functioning or unsatisfactory sexual life was related to poorer quality of life at younger age, treatment with chemotherapy, total mastectomy, emotional distress consequent on an unsatisfactory sexual life, and difficulties with partners because of sexual relationships.

## Discussion

This bibliographic review has provided an extensive list of studies that focused on quality of life in breast cancer patients. The article might be criticized on the grounds that it included every paper on the topic and that it provides more enumeration than insight. However, this was not an in-depth review but rather, as indicated in the title, a bibliographic investigation and descriptive in nature. The benefit of such an approach is that it reveals how much effort has been made in this area and shows the achievements of a journey that was started more than 30 years ago. If quality of life has now become an important part of breast cancer patients' care, it is due to all these efforts. Furthermore, this approach might help potential investigators to formulate new questions or conduct more focused studies on the topic in the future. It should be admitted that investigations of this type have limitations and are inconclusive. Since in this review the search strategy was limited to the key words 'quality of life' and 'breast cancer' in titles, perhaps many other papers also were missed even from enumeration. However, an up coming complementary review by the author will focus on these missing papers.

A number of studies that covered measurement issues and introduced instruments used to measure quality of life in breast cancer patients. Hopefully there is now sufficient evidence to use these valid instruments and to adopt the practices that are needed to assess quality of life in research or clinical settings. Since 1974, when the first study on quality of life in breast cancer patients was published, there has been quite impressive progress and improvement, indicating that measuring quality of life in breast cancer patients is both crucial and scientific. Now several valid instruments that capture quality of life dimensions in cancer patients in general and in breast cancer patients in particular are available. The EORTC QLQ-C30, EORTC QLQ-BR23, FACIT-G and FACIT-B are among the most acceptable instruments to patients and health professionals. They have been used in many studies, so it is possible to compare results between studies with similar objectives. It seems that it is time to stop developing new instruments, since there are enough valid and comprehensive measures to assess quality of life in breast cancer patients. New instruments might cause confusion and may be regarded as a waste of resources, so any such developments would need robust justification. Depending on the objectives of any single study, one might use other existing valid measures such as the Satisfaction with Life Domains Scale for Breast Cancer (SLDS-BC), which can briefly and rapidly assess quality of life across the breast cancer continuum of care [287]; the Body Image After Breast Cancer Questionnaire (BIBCQ); which is a valid measure for assessing the long-term impact of breast cancer on body image [288]; and the Fallowfield's Sexual Activity Questionnaire (FSAQ), which is a useful tool for measuring sexual activity in women with cancer [289].

There were some important technical issues that should be addressed. Some believe that if we perform complex analyses of quality of life data or if we use several instruments in a single study then we might achieve more scientific results. There is evidence that this could merely lead to misleading findings and might be a source of suffering for the patients [84]. The recommendation is to analyze data in a simple way and avoid complexity. The presentation of data should be straightforward and easy to follow; otherwise those who are critical of such findings might conclude that these are manipulations of data, or they might ask whether these numbers and statistics reflect what really happens to breast cancer patients or the clinical teams that care for them. Do these figures convey difficulties that exist in treating breast cancer patients or help to manage their symptoms?

The present review covered several topics and provided tables to indicate areas that need more attention. It appears that the most common and important disease- and treatment-related side-effects and symptoms in breast cancer patients including arm morbidity, pain, fatigue and postmenopausal symptoms, are among neglected topics. As noted by Cella and Fallowfield, recognition and management of treatment-related side-effects for breast cancer patients receiving adjuvant endocrine therapy is an important issue since such side-effects negatively affect health-related quality of life and adherences to therapy. These authors argue that adverse events constitute the main reason for non-adherence to endocrine treatment, and across all adjuvant endocrine trials regardless of the treatment, vasomotor symptoms such as hot flushes are the most common side effects. Other frequently reported side-effects such as vaginal discharge, vaginal dryness, dyspareunia, and arthralgia vary in prevalence between tamoxifen and aromatase inhibitors [290]. It has been recommended that currently in assessing quality of life in breast cancer patients priorities should be given to cognitive functioning, menopausal symptoms, body image and long-term effects of new therapies that might cause musculoskeletal and neurological side-effects [35]. In addition, sexual functioning seems important area that needs more attention, especially for younger breast cancer survivors. It is argued that younger survivors may need interventions that specifically target their needs related to menopausal symptoms and problems with relationships, sexual functioning and body image [291].

There were few qualitative studies. Since these could provide more insight into quality of life in breast cancer patients, we need more such studies to collect data and indicate how breast cancer patients interpret life after diagnosis and during and after treatment. Breast cancer survivors even might rate their quality of life more favorably than outpatients with other common medical conditions and identify many positive aspects from the cancer experience [180]. However, it is not only the study of quality of life in newly diagnosed breast cancer patients that is necessary; studying quality of life in long-term survivors is equally important. As suggested, when assessing quality of life in breast cancer patients, the stage of disease should also be considered. There are differences in quality of life between patients with non-invasive breast cancer, newly diagnosed breast cancer and advanced local breast cancer, and disease-free breast cancer survivors, women with recurrence breast cancer, and women with advanced metastatic breast cancer [292].

## Conclusion

There was quite an extensive body of the literature on quality of life in breast cancer patients. These papers have made a considerable contribution to improving breast cancer care, although their exact benefit was hard to define. However, quality of life data provided scientific evidence for clinical decision-making and conveyed helpful information concerning breast cancer patients' experiences during the course of the disease diagnosis, treatment, disease-free survival time, and recurrences; otherwise finding patient-centered solutions for evidence-based selection of optimal treatments, psychosocial interventions, patient-physician communications, allocation of resources, and indicating research priorities were impossible. It seems that more qualitative research is needed for a better understanding of the topic. In addition, issues related to the disease, its treatment side effects and symptoms, and sexual functioning should receive more attention when studying quality of life in breast cancer patients.

## Competing interests

The author declares that they have no competing interests.

## Authors' contributions

The author carried out this review and wrote the manuscript, and prepared all the tables and the additional file.

## Supplementary Material

Additional file 1 Quality of life in breast cancer patients. This is a chronological list of all papers that were published since 1974 to the end of year 2007 in the English biomedical journals. The list is organized for each year and only contains papers that used the word quality of life and breast cancer or breast carcinoma in their titles. The papers are sorted alphabetically. Click here for file
