# Supplementary material for: Health-related quality of life in breast cancer patients: A bibliographic review of the literature from 1974 to 2007
Source: J Exp Clin Cancer Res. 2008 Aug 29;27(1):32. doi: 10.1186/1756-9966-27-32 (PMC2543010; doi:10.1186/1756-9966-27-32)
Supplement: Additional file 1 — Quality of life in breast cancer patients. This is a chronological list of all papers that were published since 1974 to the end of year 2007 in the English biomedical journals. The list is organized for each year and only contains papers that used the word quality of life and breast cancer or breast carcinoma in their titles. The papers are sorted alphabetically. [file 1756-9966-27-32-S1.doc]

**Health related quality of life in breast cancer patients: a bibliographic review of the literature from 1974 to 2007**

**Ali Montazeri**

Professor in Public Health and Epidemiology

Iranian Institute for Health Sciences Research

Tehran, Iran

E-mail: [montazeri@acecr.ac.ir](mailto:montazeri@acecr.ac.ir)

**Health related quality of life in breast cancer patients: a bibliographic review of the literature from 1974 to 2007**

This is a chronological list of all papers that were reviewed. The list is organized for each year and only contains papers that used the word quality of life and breast cancer or breast carcinoma in their title. The papers are sorted alphabetically.

**2007**

1. Ahn SH, Park BW, Noh DY, Nam SJ, Lee ES, Lee MK, Kim SH, Lee KM, Park SM, Yun YH: **Health-related quality of life in disease-free survivors of breast cancer with the general population.** *Ann Oncol* 2007, 18:173-182.

2. Andritsch E, Dietmaier G, Hofmann G, Zloklikovits S, Samonigg H: **Global quality of life and its potential predictors in breast cancer patients: an exploratory study.** *Support Care Cancer* 2007, 15:21-30.

3. Awadalla AW, Ohaeri JU, Gholoum A, Khalid AO, Hamad HM, Jacob A: **Factors associated with quality of life of outpatients with breast cancer and gynecologic cancers and their family caregivers: a controlled study.** *BMC Cancer* 2007, 7:102.

4. Baumgartner KB, Fetherolf J, Reeve BB, Smith AW, Ganz PA, McTiernan A, Barbash RB: **Possible socioeconomic and ethnic disparities in quality of life in a cohort of breast cancer survivors.** *Breast Cancer Res Treat* 2007, 106:85-95.

5. Beckjord E, Campas BE: **Sexual quality of life in women with newly diagnosed breast cancer.** *J Psychosoc Oncol* 2007, 25(2): 19-36.

6. Bernhard J, Zahrieh D, Castiglione-Gertsch M, Hürny C, Gelber RD, Forbes JF, Murray E, Collins J, Aebi S, Thürlimann B, Price KN, Goldhirsch A, Coates AS, International Breast Cancer Study Group Trial VIII: **Adjuvant chemotherapy followed by goserelin compared with either modality alone: the impact on amenorrhea, hot flashes, and quality of life in premenopausal patients: the International Breast Cancer Study Group Trial VIII.** *J Clin Oncol* 2007, 25:263-270.

7. Bowen DJ, Alfano CM, McGregor BA, Kuniyuki A, Bernstein L, Meeske K,

Bulotiene G, Veseliunas J, Ostapenko V: **Quality of life of Lithuanian women with early stage breast cancer.** *BMC Public Health* 2007, 7:124.

8. Chang EW, Tsai YY, Chang TW, Tsao CJ: **Quality of sleep and quality of life in caregivers of breast cancer patient.** *Psychooncology* 2007, 16:950-955.

9. Chang JT, Chen CJ, Lin YC, Chen YC, Lin CY, Cheng AJ: **Health-related quality of life and patient satisfaction after treatment for breast cancer in northern Taiwan.** *Int J Radiat Oncol Biol Phys* 2007, 69:49-53.

10. Costanzo ES, Lutgendorf SK, Mattes ML, Trehan S, Robinson CB, Tewfik F, Roman SL: **Adjusting to life after treatment: distress and quality of life following treatment for breast cancer.** *Br J Cancer* 2007, 97:1625-1631.

11. Dagnelie PC, Pijls-Johannesma MC, Lambin P, Beijer S, De Ruysscher D, Kempen GI: **Impact of fatigue on overall quality of life in lung and breast cancer patients selected for high-dose radiotherapy.** *Ann Oncol* 2007, 18:940-944.

12. Darga LL, Magnan M, Mood D, Hryniuk WM, DiLaura NM, Djuric Z: **Quality of life as a predictor of weight loss in obese, early-stage breast cancer survivors.** *Oncol Nurs Forum* 2007, 34:86-92.

13. Dian D, Schwenn K, Mylonas I, Janni W, Friese K, Jaenicke F: **Quality of life among breast cancer patients undergoing autologous breast reconstruction versus breast conserving therapy.** *J Cancer Res Clin Oncol* 2007, 133:247-252.

14. Diel IJ: **Effectiveness of bisphosphonates on bone pain and quality of life in breast cancer patients with metastatic bone disease: a review.** *Support Care Cancer* 2007, 15:1243-1249.

15. Fatone AM, Moadel AB, Foley FW, Fleming M, Jandorf L: **Urban voices: the quality-of-life experience among women of color with breast cancer.** *Palliat Support Care* 2007, 5:115-125.

16. Franke HR, Brood-van Zanten MM, Burger CW, van der Mooren MJ, Kenemans P: **Breast cancer and climacteric complaints: weighing up risks of hormone therapy against quality of life.** *Eur J Obstet Gynecol Reprod Biol* 2007, 134:143-146.

17. Grimison PS, Stockler MR: **Quality of life and adjuvant systemic therapy for early-stage breast cancer.** *Expert Rev Anticancer Ther* 2007, 7:1123-1134.

18. Gupta D, Granick J, Grutsch JF, Lis CG: **The prognostic association of health-related quality of life scores with survival in breast cancer.** *Support Care Cancer* 2007, 15:387-393.

19. Hartmann U, Muche R, Reuss-Borst M: **Effects of a step-by-step inpatient rehabilitation programme on quality of life in breast cancer patients. A prospective randomised study.** Onkologie 2007, 30:177-182.

20. Hopwood P, Haviland J, Mills J, Sumo G, M Bliss J, START Trial Management Group: **The impact of age and clinical factors on quality of life in early breast cancer: an analysis of 2208 women recruited to the UK START Trial (Standardisation of Breast Radiotherapy Trial).** *Breast* 2007, 16:241-251.

21. Janz NK, Mujahid M, Chung LK, Lantz PM, Hawley ST, Morrow M, Schwartz K, Katz SJ: **Symptom experience and quality of life of women following breast cancer treatment.** *J Womens Health* 2007, 16:1348-1361.

22. Karamouzis MV, Ioannidis G, Rigatos G: **Quality of life in metastatic breast cancer patients under chemotherapy or supportive care: a single-institution comparative study.** *Eur J Cancer Care* 2007, 16:433-438.

23. Kenne Sarenmalm E, Ohlén J, Jonsson T, Gaston-Johansson F. **Coping with recurrent breast cancer: predictors of distressing symptoms and health-related quality of life.** *J Pain Symptom Manage* 2007, 34:24-39.

24. Kim SJ, Yi CH, Kwon OY: **Effect of complex decongestive therapy on edema and the quality of life in breast cancer patients with unilateral leymphedema.** *Lymphology* 2007, 40:143-151.

25. Lee MK, Son BH, Hwang SY, Han W, Yang JH, Lee S, Yun YH: **Factors affecting health-related quality of life in women with recurrent breast cancer in Korea.** *Qual Life Res* 2007, 16:559-569.

26. Lidgren M, Wilking N, Jönsson B, Rehnberg C: **Health related quality of life in different states of breast cancer.** *Qual Life Res* 2007, 16:1073-1081.

27. Lu W, Cui Y, Zheng Y, Gu K, Cai H, Li Q, Zheng W, Shu XO: **Impact of newly diagnosed breast cancer on quality of life among Chinese women.** *Breast Cancer Res Treat* 2007, 102:201-210.

28. Mehnert A, Scherwath A, Schirmer L, Schleimer B, Petersen C, Schulz-Kindermann F, Zander AR, Koch U: **The association between neuropsychological impairment, self-perceived cognitive deficits, fatigue and health related quality of life in breast cancer survivors following standard adjuvant versus high-dose chemotherapy.** *Patient Educ Couns* 2007, 66:108-118.

29. Meneses KD, McNees P, Loerzel VW, Su X, Zhang Y, Hassey LA: **Transition from treatment to survivorship: effects of a psychoeducational intervention on quality of life in breast cancer survivors.** *Oncol Nurs Forum* 2007, 34:1007-1016.

30. Milne HM, Guilfoyle A, Gordon S, Wallman KE, Courneya KS: **Personal accounts of exercise and quality of life from the perspective of breast cancer survivors.** *Qual Life Res* 2007, 16:1473-1481.

31. Milne HM, Gordon S, Guilfoyle A, Wallman KE, Courneya KS: **Association between physical activity and quality of life among Western Australian breast cancer survivors.** *Psychooncology* 2007, 16:1059-1068.

32. Moadel AB, Shah C, Wylie-Rosett J, Harris MS, Patel SR, Hall CB, Sparano JA: **Randomized controlled trial of yoga among a multiethnic sample of breast cancer patients: effects on quality of life.** *J Clin Oncol* 2007, 25:4387-4395.

33. Moore HC: **Impact on quality of life of adjuvant therapy for breast cancer**. *Curr Oncol Rep* 2007, 9:42-46.

34. Ogce F, Ozkan S, Baltalarli B: **Psychosocial stressors, social support and socio-demographic variables as determinants of quality of life of Turkish breast cancer patients.** *Asian Pac J Cancer Prev* 2007, 8:77-82.

35. Ohsumi S, Shimozuma K, Kuroi K, Ono M, Imai H: **Quality of life of breast cancer patients and types of surgery for breast cancer: current status and unresolved issues.** *Breast Cancer* 2007, 14:66-73.

36. Parker PA, Youssef A, Walker S, Basen-Engquist K, Cohen L, Gritz ER, Wei QX, Robb GL: **Short-term and long-term psychosocial adjustment and quality of life in women undergoing different surgical procedures for breast cancer.** *Ann Surg Oncol* 2007, 14:3078-3089.

37. Perry S, Kowalski TL, Chang CH: **Quality of life assessment in women with breast cancer: benefits, acceptability and utilization.** *Health Qual Life Outcomes* 2007, 5: 24.

38. Peuckmann V, Ekholm O, Rasmussen NK, Møller S, Groenvold M, Christiansen P, Eriksen J, Sjøgren P. **Health-related quality of life in long-term breast cancer survivors: nationwide survey in Denmark.** *Breast Cancer Res Treat* 2007, 104:39-46.

39. Richardson LC, Wang W, Hartzema AG, Wagner S: **The role of health-related quality of life in early discontinuation of chemotherapy for breast cancer.** *Breast J* 2007, 13:581-587.

40. Robb C, Haley WE, Balducci L, Extermann M, Perkins EA, Small BJ, Mortimer J: **Impact of breast cancer survivorship on quality of life in older women.** *Crit Rev Oncol Hematol* 2007, 62:84-91.

41. Rozenberg S, Antoine C, Carly B, Pastijn A, Liebens F: Improving quality of life after breast cancer: prevention of other diseases. Menopause Int 2007, 13:71-74.

42. Saatci E, Akin S, Akpinar E: **Do the unmet needs affect the quality of life in breast cancer patients?** *West Indian Med J* 2007, 56:253-257.

43. Scott C, Suh J, Stea B, Nabid A, Hackman J: **Improved survival, quality of life, and quality-adjusted survival in breast cancer patients treated with efaproxiral (Efaproxyn) plus whole-brain radiation therapy for brain metastases.** *Am J Clin Oncol* 2007, 30:580-587.

44. Tercyak KP, Peshkin BN, Brogan BM, DeMarco T, Pennanen MF, Willey SC, Magnant CM, Rogers S, Isaacs C, Schwartz MD: **Quality of life after contralateral prophylactic mastectomy in newly diagnosed high-risk breast cancer patients who underwent BRCA1/2 gene testing.** *J Clin Oncol* 2007, 25:285-291.

45. Titeca G, Poot F, Cassart D, Defays B, Pirard D, Comas M, Vereecken P, Verschaevec V, Simon P, Heenen M: **Impact of cosmetic care on quality of life in breast cancer patients during chemotherapy and radiotherapy: an initial randomized controlled study.** *J Eur Acad Dermatol Venereol* 2007, 21:771-776.

46. Vallance JK, Courneya KS, Plotnikoff RC, Yasui Y, Mackey JR: **Randomized controlled trial of the effects of print materials and step pedometers on physical activity and quality of life in breast cancer survivors.** *J Clin Oncol* 2007, 25:2352-2359.

47. van Gestel YR, Voogd AC, Vingerhoets AJ, Mols F, Nieuwenhuijzen GA, van Driel OJ, van Berlo CL, van de Poll-Franse LV: **A comparison of quality of life, disease impact and risk perception in women with invasive breast cancer and ductal carcinoma in situ.** *Eur J Cancer* 2007, 43:549-556.

48. Waldmann A, Pritzkuleit R, Raspe H, Katalinic A: **The OVIS study: health related quality of life measured by the EORTC QLQ-C30 and -BR23 in German female patients with breast cancer from Schleswig-Holstein.** *Qual Life Res* 2007, 16:767-776.

49. Wan C, Zhang D, Yang Z, Tu X, Tang W, Feng C, Wang H, Tang X: **Validation of the simplified Chinese version of the FACT-B for measuring quality of life for patients with breast cancer.** *Breast Cancer Res Treat* 2007, 106:413-418.

50. Wan C, Tang X, Tu XM, Feng C, Messing S, Meng Q, Zhang X: **Psychometric properties of the simplified Chinese version of the EORTC QLQ-BR53 for measuring quality of life for breast cancer patients.** *Breast Cancer Res Treat* 2007, 105:187-193.

51. Wong WS, Fielding R: **Change in quality of life in Chinese women with breast cancer: changes in psychological distress as a predictor.** *Support Care Cancer* 2007, 15:1223-1230.

**2006**

1. Amado F, Lourenco MT, Deheinzelin D: **Metastatic breast cancer: do current treatments improve quality of life? A prospective study.** *Sao Paulo Med J* 2006, 124: 203-207.

2. Ankerst DP, Engle J: **Status of quality of life in breast cancer research before, during and beyond treatment.** *Epert Rev Pharmacoecon Outcomes Res* 2006, 6: 207-214.

3. Antoni MH, Lechner SC, Kazi A, Wimberly SR, Sifre T, Urcuyo KR, Phillips K, Gluck S, Carver CS: **How stress management improves quality of life after treatment for breast cancer.** *J Consult Clin Psychol* 2006, 74: 1143-1152.

4. Arndt V, Stegmaier C, Ziegler H, Brenner H: **A population-based study of the impact of specific symptoms on quality of life in women with breast cancer 1 year after diagnosis.** *Cancer* 2006, 107: 2496-2503.

5. Avis NE, Foley KL: **Evaluation of the Quality of Life in Adult Cancer Survivors (QLACS) scale for long term cancer survivors in a sample of breast cancer survivors.** *Health Qual Life Outcomes* 2006, 4: 92.

6. Bozcuk H, Artac M, Kara A, Ozdogan M, Sualp Y, Topcu Z, Karaagacli A, Yildiz M, Savas B: **Does music exposure during chemotherapy improve quality of life in early breast cancer patients? A pilot study.** *Med Sci Monit* 2006, 12: CR200-205.

7. Byar KL, Berger AM, Bakken SL, Cetak MA: **Impact of adjuvant breast cancer chemotherapy on fatigue, other symptoms and quality of life.** *Oncol Nurs Forum* 2006, 33: E18-E26.

8. Carlsson M, Arman M, Backman M, Flatters U, Hatschek T, Hamrin E: **A five-year follow-up of quality of life in women with breast cancer in anthroposophic and conventional care.** *Evidence Based Complement Alternat Med* 2006, 3: 523-531.

9. Carver CS, Smith RG, Petronis VM, Antoni MH: **Quality of life among long-term survivors of breast cancer: different types of antecedents predict different class of outcomes.** *Psycho-Oncol* 2006, 15: 749-758.

10. Cella D, Fallowfield L, Barker P, Cuzick J, Locker G, Howell A: **Quality of life of postmenopausal women in the ATAC (Arimidex, tamoxifen, alone or in combination) trial after completion of 5 years’ adjuvant treatment for early breast cancer.** *Breast Cancer Res & Treat* 2006, 100: 273-284.

11. Cheema BSB, Gaul CA: **Full-body exercise training improves fitness and quality of life in survivors of breast cancer.** *J Strenght Condition Res* 2006, 20: 14-21.

12. Claus EB, Petruzella S, Carter D, Kasi S: **Quality of life for women diagnosed with breast carcinoma in situ.** *J Clin Oncol* 2006, 24: 4875-4881.

13. Cui Y, Shu XO, Gao YT, Cai H, Tao MH, Zheng W: **Association of ginseng use with survival and quality of life among breast cancer patients.** *Am J Epidemiol* 2006, 163: 645-653.

14. Fallowfield LJ, Bliss JM, Porter LS, Price MH, Snowdon CF, Jones SE, Coobes RC, Hall E: **Quality of life in the intergroup exemestane study: a randomized trial of exemestane versus continued tamoxifen after 2 to 3 years of tamoxifen in postmenopausal women with primary breast cancer.** *J Clin Oncol* 2006, 24: 910-917.

15. Fleissig A, Fallowfield LJ, Langridge CI, Johnson L, Newcombe RG, Dixon JM, Kissin M, Mansel RE: **Post-operative arm morbidity and quality of life. Results of the ALMANAC randomized trial comparing sentinel node biopsy with standard axillary treatment in the management of patients with early breast cancer.** *Breast Cancer Res Treat* 2006, 95: 279-293.

16. Geiger AM, West CN, Nekhlyudov L, Herrinton LJ, Liu IA, Altschuler A, Rolink SJ, Harris EL, Green SM, Elmore JG, Emmons KM, Fletcher SW: **Contentment with quality of life among breast cancer survivors with and without contralateral prophylactic mastectomy.** *J Clin Oncol* 2006, 24: 1350-1356.

17. Giese-Davis J, Bliss-Isberg C, Carson K, Star P, Donaghy J, Cordova MJ, Stevens N, Wittenberg L, Batten C, Spiegel D: **The effect of peer counseling on quality of life following diagnosis of breast cancer: an observational study.** *Psycho-Oncol* 2006, 15: 1014-1022.

18. Grabsch B, Clarke DM, Love A, McKenzie DP, Snyder RD, Bloch S, Smith G, Kissane DW: **Psychological morbidity and quality of life in women with advanced breast cancer: a cross-sectional survey.** *Palliat Support Care* 2006, 4: 47-56.

19. Groenvold M, Fayers PM, Petersen MA, Mouridsen HT: **Chemotherapy versus ovarian ablation as adjuvant therapy for breast cancer: impact on health-related quality of life in a randomized trial.** *Breast Cancer Res Treat* 2006, 98: 275-284.

20. Gupta P, Sturdee DW, Pallin SL, Majumder K, Fear R, Marshall T, Paterson I: **Menopausal symptoms in women treated for breast cancer; the prevalence and severity of symptoms and their perceived effects on quality of life.** *Climacteric* 2006, 9: 49-58.

21. Hanser SB, Bauer-Wu S, Kubicek L, Healey M, Manola J, Hernandez M, Bunnell C: **Effects of a music therapy intervention on quality of life and distress in women with metastatic breast cancer.** *J Soc Integr Oncol* 2006, 4: 116-124.

22. Heidrich SM, Egan JJ, Hengudomsub P, Randolph SM: **Symptoms, symptom beliefs, and quality of life of older breast cancer survivors: a comparative study.** *Oncol Nurs Forum* 2006, 33: 315-22.

23. Herrero F, Balmer J, San Juan AF, Foster C, Fleck SJ, Perez M, Canete S, Earnest CP, Lucia A: **Is cardiorespiratory fitness related to quality of life in survivors of breast cancer*?*** *J Strength Condition Res* 2006, 20: 535-540.

24. Hurria A, Zuckerman E, Panageas KS, Fornier M, D’Andrea G, Dang C, Moasser M, Robson M, Seidman A, Currie V, Van Poznak C, Theodoulou M, LachsMS, Hudis C: **A prospective, longitudinal study of the functional status and quality of life of older patients with breast cancer receiving adjuvant chemotherapy.** *J Am Geri Soc* 2006, 54: 1119-1124.

25. Kayl AE, Meyers CA: **Side-effects of chemotherapy and quality of life in overian and breast cancer patients.** *Current Opinion in Obstetric & Gynecology* 2006, 18: 24-28.

26. Kim J, Ashing-Giwa KT, Singer MK, Tejero JS: **Breast cancer among Asian Americans: is acculturation related to health-related quality of life?** *Oncol Nurs Forum* 2006, 33: E90-E99.

27. Knobf MT: **The influence of endocrine effects of adjuvant therapy on quality of life outcomes in younger breast cancer survivors.** *Oncologist* 2006, 11: 96-110.

28. Land SR, Wickerham DL, Costantino JP, Ritter MW, Vogel VG, Lee MK, Pajon ER, Wade JLIII, Dakhil S, Lockhart JB, Wolmark N, Ganz PA: **Patient reported symptoms and quality of life during treatment with tamoxifen or raloxifene for breast cancer prevention: the NSABP study of tamoxifen and raloxifene (STAR) P-2 trial.** *JAMA* 2006, 295: 2742-2751.

29. Liu J, Tu D, Dancey J, Reyno L, Pritchard KI, Pater J, Seymour LK: **Quality of life analyses in a clinical trial of DPPE (tesmilifene) plus doxorubicin versus doxorubicin in patients with advanced or metastatic breast cancer: NCIC CTG Trial MA 19.** *Breast Cancer Res Treat* 2006, 100: 263-271.

30. Malinovszky KM, Gould A, Foster E, Cameron D, Humphreys A, Crown J, Leonard RC: **Quality of life and sexual function after high-dose or conventional chemotherapy for high-risk breast cancer.** *Br J Cancer* 2006, 95: 1626-1631.

31. Martin M, Lluch a, Segui MA, Ruzi A, Ramos M, adrover E, Rodriguez-Lescure A, Grosse R, Calvo L, Fernandez-Chacon C, Roset M, Anton A, Isla D, del Prado PM, Iglesias L, Zaluski J, Arcusa A, Lopez-Vega JM, Munoz M, Mel JR: **Toxicity and health-related quality of life in breast patients receiving adjuvant docetaxel, doxorubicin, cyclophosphamide (TAC) or 5-fluorouracil, doxorubicin and cyclophosphamide (FAC): impact of adding primary prophylactic granulocyte-colony stimulating factor to the TAC regimen.** *Annal Oncol* 2006, 17: 1205-1212.

32. Massacesi C, Sabbatini E, Rocchi MB, Zepponi L, Rossini S, Pilone A, Burattini L, Pezzoli M: **Effects of switching from tamoxifen to anastrozole on tamoxifen-related endocrine symptoms and quality of life.** *Am J Cancer* 2006, 5: 433-440.

33. Milne RJ, heaton-Brown KH, Hansen P, Thomas D, Harvey V, Cubitt A: **Quality-of-life valuations of advanced breast cancer by New Zealand women.** *Pharmacoeconomics* 2006, 24: 281-292.

34. Morgan PD, Gaston-Johansson F, Mock V: **Spritual well-being, religious coping, and the quality of life of African American breast cancer treatment: a pilot study.** *ABNF J* 2006, 17: 73-77.

35. Neyt M, Albrecht J: **The long-term evaluation of quality of life for disease-free breast cancer survivors: a comparative study in Belgium.** *J Psychosoc Oncol* 2006, 24: 89-123.

36. Ohira T, Schmitz KH, Ahmed RL, Yee D: **Effects of weight training on quality of life in recent breast cancer survivors: the weight training for breast cancer survivors (WTBS) study.** *Cancer* 2006, 106: 2076-2083.

37. Pandey M, Thomas BC, Ramdas K, Ratheesan K: **Early effect of surgery on quality of life in women with operable breast cancer.** *Jap J Clin Oncol* 2006, 36: 468-472.

38. Pyszel A, Malyszezak K, Pyszel K, andrzejak R, Szuba A: **Disability, psychological distress and quality of life in breast cancer survivors with arm lymphedema.** *Lymphology* 2006, 39: 185-192.

39. Rietman JS, Geertzen JH, Hoekstra HJ, Baas P, Dolsma WV, de Vries J, Groothoff JW, Eisma WH, Dijkstra PU: **Long term treatment related upper limb morbidity and quality of life after sentinel lymph node biopsy for stage I or II breast cancer.** *Eur J Surg Oncol* 2006, 32: 148-152.

40. Roberts J, Morden L, MacMath S, Massie K, Olivotto IA, Parker C, Hayashi A: **The quality of life of elderly women who underwent radiofrequency ablation to treat breast cancer.** *Qual Health Res* 2006, 16: 762-772.

41. Round T, Hayes SC, Newman B: **How do recovery advice and behavioral characteristics influence upper-body function and quality of life among women 6 months after breast cancer diagnosis?** *Support Care Cancer* 2006, 14: 22-29.

42. Semiglazov VF, Stepula VV, Dudov A, Schnitker J, Mengs U: **Quality of life is improved in breast cancer patients by Standardised Mistletoe Extract PS76A2 during chemotherapy and follow-up: a randomized, placebo-controlled, double-blind, multicentre clinical trial.** *Anticancer Res* 2006, 26: 1519-1529.

43. Shim EJ, Mehnert A, Koyama A, Cho SJ, Inui H, Paik NS, Koch U: **Health-related quality of life in breast cancer: a cross-cultural survey of German, Japanese, and South Korean patients*.*** *Breast Cancer Res Treat* 2006, 99: 341-350.

44. Sutton LB, Erlen JA: **Effects of mutual dyad support on quality of life in women with breast cancer.** *Cancer Nurs* 2006, 29: 488-498.

45. Wagner CD, Bigatti SM, Storniolo AM: **Quality of life of husbands of women with breast cancer.** *Psycho-Oncol* 2006, 15: 109-120.

46. Wayne SJ, Baumgartner K, Baumgartner RN, Bernstein L, Bowen DJ, Ballard-Barbash R: **Diet quality is directly associated with quality of life in breast cancer survivors.** *Breast Cancer Res Treat* 2006, 96: 227-232.

47. Wonghongkul T, Dechaprom N, Phumivichuvate L, Losawatkul S: **Uncertainty appraisal coping and quality of life in breast cancer survivors.** *Cancer Nurs*, 29: 250-257.

48. Yen JY, Ko CH, Yen CF, Yang MJ, Wu CY, Juan CH, Hou MF: **Quality of life, depression, and stress in breast cancer women outpatients receiving active therapy in Taiwan*.*** *Psychiatry & Clinical Neurosciences* 2006, 60: 147-153.

**2005**

1. Ahles TA, Saykin AJ, Furstenberg CT, Cole B, Mott LA, Ttius-Ernstoff L, Skalla K, Bakitas M, Silberfarb PM: **Quality of life of long-term survivors of breast cancer and lymphoma treated with standard-dose chemotherapy or local therapy.** *J Clin Oncol* 2005, 23: 4399-4405.

2. Aranda S, Schofield P, Weih L, Yates P, Milne D, Faulkner R, Voudouris N: **Mapping the quality of life and unmet needs of urban women with metastatic breast cancer.** *Eur J Cancer Care* 2005, 4: 211-222.

3. Arndt V, Merx H, Stegmaier C, Ziegler H, Brenner H: **Persistence of restrictions in quality of life from the first to the third year after diagnosis in women with breast cancer*.*** *J Clin Oncol* 2005, 23: 4945-4953.

4. Avis NE, Crawford S, Manuel J: **Quality of life among younger women with breast cancer.** *J Clin Oncol* 2005, 23: 3322-3330.

5. Back M, Ahern V, Delaney G, Graham P, Steigler A, Wratten C: **Absence of adverse early quality of life outcomes of radiation therapy in breast conservation therapy for early breast cancer.** *Australasian Radiology* 2005, 49: 39-43.

6. Badger T, Segrin C, Meek P, Lopez AM, Bonham E, Sieger A: **Telephone interpersonal counseling with women with breast cancer: symptom management and quality of life.** *Oncol Nurs Forum* 2005, 32: 273-279.

7. Barranger E, Dubernard G, Fleurence J, Antoine M, darai e, Uzan S: **Subjective morbidity and quality of life after sentinel node biopsy and axillary lymph node dissection for breast cancer.** *J Sur Oncol* 2005, 92: 17-22.

8. Bottomley A, Therasse P, Piccart M, Efficace F, Coens C, Gotay C, Welnicka-Jaskiewicz M, Mauriac L, Dyczka J, Cufer T, Lichinitser MR, Schornagel JH, Bonnefoi H, Shepherd L: **Health-related quality of life in survivors of locally advanced breast cancer: an international randomized controlled phase III trial.** *Lancet Oncol* 2005, 6: 287-294.

9. Burckhardt CS, Carol S, Jones KD. **Effects of chronic widespread pain on the health status and quality of life of women after breast cancer surgery.** *Health Qual Life Outcomes* 2005, 3: 30.

10. Chang J, Couture F, Young S, McWatters K, Lau CY: **Weekly epoetin alfa maintains hemoglobin, improves quality of life, and reduces transfusion in breast cancer patients receiving chemotherapy.** *J Clin Oncol* 2005, 23: 2597-25605.

11. Conde DM, Pinto-Neto AM, Cabello C, Santos-Sa D, Costa-Paiva L, Martinze EZ: **Menopause symptoms and quality of life in women aged 45 to 65 years with and without breast cancer.** *Menopause* 2005, 12: 436-443.

12. Conden DM, Pinto-Neto AM, Cabello C, Santos-Sa D, Costa-Paiva L, Martinez EZ: **Quality of life in Brazilian breast cancer survivors age 45-65 years: associated factors.** *Breast J* 2005, 11: 425-432.

13. Conner-Spady BL, Cumming C, Nabholtz JM, Jacobs P, Stewart D: **A longitudinal prospective study of health-related quality of life in breast cancer patients following high-dose chemotherapy with autologous blood stem cell transplantation.** *Bone Marrow Transplantation* 2005; 36: 251-259.

14. Deshields T, Tibbs T, Fan MY, Bayer L, Taylor M, Fisher E: **Ending treatment: the course of emotional adjustment and quality of life among breast cancer survivors immediately following radiation therapy.** *Support Care Cancer* 2005, 13: 1018-1026.

15. Elder EE, Brandberg Y, Bjorklund T, rylander r, Lagerren J, Jurell G, Wickman M, Sandelin K. **Quality of life and patient satisfaction in breast cancer patients after immediate breast reconstruction: a prospective study.** *Breast* 2005, 14: 201-208.

16. Elkin EB, Weinstein MC, Kuntz KM, Bunnell CA, Weeks JC: **Adjuvant ovarian suppression versus chemotherapy for premenopausal, hormone-responsive breast cancer: quality of life and efficacy tradeoffs.** *Breast Cancer Res Treat* 2005, 93: 25-34.

17. Fehlauer F, Tribius S, Mehnert A, Rades D: **Health-related quality of life in long term breast cancer survivors treated with breast conserving therapy: impact of age at therapy**. *Breast Cancer Res Treat* 2005, 92: 217-222.

18. Galalae RM, Michel J, Siebmann JU, Kuchler T, Eilf K, Kimmig B: **Significant negative impact of adjuvant chemotherapy on health-related quality of life (HR-QoL) in women with breast cancer treated by conserving surgery and postoperative 3-D radiotherapy.** **A prospective measurement.** *Strahlenther Onkol* 2005, (Strahlentherapie und Onkologie; 181: 645-651.

19. Golden-Kreutz DM, Thornton LM, Wells-Di GS, Frierson GM, Jim HS, Carpenter KM, Shelby RA, Andersen BL: **Traumatic stress, perceived global stress, and life events: prospectively predicting quality of life in breast cancer patients.** *Health Psychology* 2005; 24: 288-296.

20. Gordon LG, Battistutta D, Scuffham P, Tweeddale M, Newman B. **The impact of rehabilitation support services on health-related quality of life for women with breast cancer.** *Breast Cancer Res Treat* 2005, 93: 217-226.

21. Hudis CA, Vogel CL, Gralow JR, Williams D: **Weekly epoetin alfa during adjuvant chemotherapy for breast cancer: effect on hemoglobin levels and quality of life.** *Clin Breast Cancer* 2005, 6: 132-142.

22. Janz NK, Mujahid M, Lantz PM, Fagerlin A, Salem B, Morrow M, Deapen D, Katz SJ: **Population-based study of the relationship of treatment and sociodemographics on quality of life for early stage breast cancer.** *Qual Life Res* 2005, 14: 1467-1479.

23. Kendall AR, Mahue-Giangreco M, Carpenter CL, Ganz PA, Bernstein L: **Influence of exercise activity on quality of life in long term breast cancer survivors.** *Qual Life Res* 2005, 14: 361-371.

24. Laidlaw T, Bennett BM, Dwivedi P, Naito A, Gruzellier J: **Quality of life and mood changes in metastatic breast cancer after training in self-hypnosis or johrei: a short report.** *Contemp Hypn* 2005, 22: 84-93.

25. Lehto US, Ojanen M, Kellokumpu-Lehtinen P. **Predictor of quality of life in newly diagnosed melanoma and breast cancer patients.** *Annal Oncol* 2005, 16: 805-816.

26. Lopez EDS, Eng E, Randall-David E, Robinson N: **Quality-of-life concerns of African American breast cancer survivors within rural North Carolina: blending the techniques of photovoice and grounded theory.** *Qual Health Res* 2005, 15: 99-115.

27. Luini A, Gatti G, Zurrida S, Galimberti V, Paganelli G, Naninato P, Caldarella P, Rotmensz N, Winnikow E, Viale G: **The sentinel lymph node biopsy under local anesthesia in breast carcinoma: experience of the European Institute of Oncology and impact on quality of life.** *Breast Cancer Res Treat* 2005, 89: 69-74.

28. Manning-Walsh J: **Social support as a mediator between symptom distress and quality of life in women with breast cancer.** *J Obstetric, Gyneocologic Neonatal Nurs* 2005, 34: 482-493.

29. Manning-Walsh J: **Spiritual struggle: effect on quality of life and life satisfaction in women with breast cancer*.*** *J Holistic Nurs* 2005, 23: 120-140.

30. Marcy PY, Magne N, Castadot P, Bailet C, Macchiavello JC, Namer M, Gallard JC: **Radiological and surgical placement of port devices: a 4-year institutional analysis of procedure performance, quality of life and cost in breast cancer patients.** *Breast Cancer Res Treat* 2005, 92: 61-67.

31. Mills PJ, Parker B, Dimsdale JE, Sadler GR, Ancoli-Israel S: **The relationship between fatigue and quality of life and inflammation during anthracyline-based chemotherapy in breast cancer.** *Biological Psychology* 2005, 69: 85-96.

32. Mols F, Vingerthoets AJ, Coebergh JW, van de Poll-Franse LV: **Quality of life among long-term breast cancer survivors: a systematic review.** *Eur J Cancer* 2005, 41: 2613-2619.

33. Northouse L, Kershaw T, Mood D, Schafenacker A: **Effects of a family intervention on the quality of life of women with recurrent breast cancer and their family caregivers.** *Psycho-Oncol* 2005, 14: 478-491.

34. Okamura M, Yamavaki S, Akechi T, Taniguchi K, Uchitomi Y: **Psychiatric disorders following first breast cancer recurrence: prevalence, associated factors and relationship to quality of life.** *Jap J Clin Oncol* 2005, 35: 302-309.

35. Pandey M, Thomas BC, SreeRekha P, Ramdas K, Ratheesan K, Parameswaran S, Mathew BS, Rajan B: **Quality of life determinants in women with breast cancer undergoing treatment with curative intent.** *World J Surg Oncol* 2005, 3: 63.

36. Parmar V, Badwe RA, Hawaldar R, Rayabhattanavar S, Varghese A, Sharma R, Mittra I: **Validation of EORTC quality-of-life questionnaire in Indian women with operable breast cancer.** *Natl Med J India* 2005, 18: 172-177.

37. Pandey M, Thomas BC, SreeRekha P, Ramdas K, Retheesan K, Parameswaran S, Mathew BS, Rajan B: **Quality of life determinants in women with breast undergoing treatment with curative intent.** *World J Surg Oncol* 2005, 3: 63.

38. Peppercorn J, Herndon II J, Kornblith AB, Peters W, Ahles T, Vredenburgh J, Schwartz G, Shpall E, Hurd DD, Holland J, Winer E: **Quality of life among patients with stage II and III breast carcinoma randomized to receive high-dose chemotherapy with autologous bone marrow support or intermediate-dose chemotherapy: results from cancer and Leukemia group B 9066.** *Cancer* 2005, 104: 1580-1589.

39. Ransom S, Jacobsen PB, Schmidt JE, Andrykowski MA: **Relationship of problem-focused coping strategies to changes in quality of life following treatment for early stage breast cancer.** *J Pain Symptom Management* 2005, 30: 243-253.

40. Ridner SH: **Quality of life and a symptom cluster associated with breast cancer treatment-related lymphedema.** *Support Care Cancer* 2005; 13: 904-911.

41. Roth RS, Lowery JC, Davis J, Wilkins E: **Quality of life and affective distress in women seeking immediate versus delayed breast reconstruction after mastectomy for breast cancer.** *Plastic & Reconstruction Surgery* 2005, 116: 993-1002.

42. Sandel SL, Judge JO, Landry N, Faria L, Oullette R, Majczak M. **Dance and movement program improves quality-of-life measures in breast cancer survivors.** *Cancer Nurs* 2005, 28: 301-309.

43. Schou I, Ekeberg O, Sandvik L, Hjermstad MJ, Ruland CM: **Multiple predictors of health-related quality of life in early stage breast cancer. Data from a year follow-upstudy compared with the general population.** *Qual Life Res* 2005, 14: 1813-1823.

44. Schultz PN, Klein MJ, Beck ML, Stava C, Sellin RV: **Breast cancer: relationship between menopausal symptoms, physiologic health effects of cancer treatment and physical constraints on quality of life in long-term survivors.** *J Clin Nurs* 2005, 14: 204-211.

45. Segrin C, Badger TA, Meek P, Lopez AM, Bonham E, Sieger A: **Dyadic interdependence on affect and quality of life trajectories among women with breast cancer and their partners.** *J Soc Personal Relationships* 2005, 22: 673-689.

46. Strauss-Blasche G, Gnad E, Ekmekcioglu C, Hladschik B, Marktl W: **Combined inpatient rehabilitation and spa therapy of breast cancer patients: effects on quality of life and CA 15-3**. *Cancer Nurs* 2005; 28: 390-398.

47. Sun A, Wong-Kim E, Stearman S, Chow EA: **Quality of life in Chinese patients with breast cancer.** *Cancer* 2005, 104 (Suppl. 12): 2952-2954.

48. Thornton AA, Madlensky L, Flatt SW, Kaplan RM, Pierce JP: **The impact of a second breast cancer diagnosis on health related quality of life.** *Breast Cancer Res Treat* 2005, 92: 25-33.

49. Tong DK, Cheng CW, Ching CS, Ngor WL, Chow LW: **Phase II study of ‘all-oral’ regimen of capecitabine, idarubicin and cyclophosphamide for metastatic breast cancer: safety, efficacy and quality of life.** *Oncology* 2005, 68: 520-525.

50. Wardley A, Davidson N, Barrett-Lee P, Hong A, Mansi J, Dodwell D, Murphy R, Mason T, Cameron D: **Zoledronic acid significantly improves pain scores and quality of life in breast cancer patients with bone metastases: a randomized, crossover study of community vs hospital bisphosphonate administration.** *Br J Cancer* 2005, 92: 1869-1876.

51. Wilson RW, Hutson LM, Vanstry D: **Comparing of 2 quality of life questionnaires in women treated for breast cancer: the RAND 36-Item Health Survey and the Functional Living Index-Cancer.** *Physical Therapy* 2005, 85: 851-860.

52. Wong-Kim E, Sun A, Merighi JR, Chow EA: **Understanding quality of life issues in Chinees women with breast cancer: a qualitative investigation.** *Cancer Control* 2005, 12 (Suppl. 2): 6-12.

53. Yoo HJ, Ahn SH, Kim SB, Kim WK, Han OS: **Efficacy of progressive muscle relaxation training and guided imagery in reducing chemotherapy side effects in patients with breast cancer and in improving their quality of life.** *Support Care Cancer* 2005, 13: 826-833.

**2004**

1. Albert US, Koller M, Wagner U, Schulz KD: **Survival chances and psychological aspects of quality of life in patients with localized early stage of breast cancer.** *Inflammation Res* 2004, 53 (Suppl. 2): S136-S141.

2. Arndt V, Merx H, Sturmer T, Stegmaier C, Ziegler H, Brenner H: **Age-specific detriments to quality of life among breast cancer patients one year after diagnosis.** *Eur J Cancer* 2004, 40: 673-680.

3. Badger TA, Braden CJ, Mishel MH, Longman A: **Depression burden, psychological adjustment, and quality of life in women with breast cancer: patterns over time.** *Res Nurs Health* 2004, 27: 19-28.

4. Bardwell WA, Major JM, Rock CL, Newman VA, Thomson CA, Chilton JA, Dimsdale JE, Pierce JP: **Health-related quality of life in women previously treated for early-stage breast cancer.** *Psycho-Oncol* 2004, 13: 595-604.

5. Bernhard J, Zahrieh D, Coats AS, Gelber RD, Castiglione-Gertsch M, Murray E, Forbes JF, Perey L, Collins J, Snyder R, Rudenstam CM, Crivellari D, Veronesi A, Thurlimann B, Fey MF, Price KN, Goldhirsch A, Hurny C: **Quantifying trade-offs: quality of life and quality-adjusted survival in a randomized trial of chemotherapy in postmenopausal patients with lymph node-negative breast cancer.** *Br J Cancer* 2004, 91: 1893-1901.

6. Bloom JR, Stewart SL, Chang S, Banks PJ: **Then and now: quality of life of young breast cancer survivors.** *Psycho-Oncol* 2004, 13: 147-160.

7. Body JJ, Diel IJ, Bell R, Pecherstorfer M, Lichinitser MR, Lazarev AF, Tripathy D, Bergstrom B: **Oral ibandronate improves bone pain and preserves quality of life in patients with skeletal metastases due to breast cancer.** *Pain* 2004, 111: 306-312.

8. Bottomely A, Biganzoli L, Cufer T, Coleman RE, Coens C, Efficace F, Calvert HA, Gamucci T, Twelves C, Fargeot P, Piccart M: **Randomized controlled trial investigating short-term health-related quality of life with doxorubicin and paclitaxel versus doxorubicin and cyclophosphamide as first-line chemotherapy in patients with metastatic breast cancer: European Organization for Research and Treatment of Cancer Breast Cancer Group, Investigational Drug Branch for Breast Cancer and the New Drug Development Group Study.** *J Clin Oncol* 2004, 22: 257-286.

9. Brandberg Y, Arver B, Lindblom A, Sandelin K, Wickman M, Hall P: **Preoperative psychological reactions and quality of life among women with an increased risk of breast cancer who are considering a prophylactic mastectomy.** *Eur J Cancer* 2004, 40: 365-374.

10. Carlson LE, Speaca M, Patel KD, Goodey E: **Mindfulness-based stress reduction in relation to quality of life, mood, symptoms of stress and levels of cortisol, dehydroepiandrosterone sulfate (DHEAS) and melatonin in breast and prostate cancer outpatients.** *Psychoneuroendocrinology* 2004, 29: 448-474.

11. Carlsson M, Arman M, Backman M, Flatters U, Hatschek T, Hamrin E: **Evaluation of quality of life/life satisfaction in women with breast cancer.** *Acta Oncol* 2004, 43: 27-34.

12. Casso DB, Buist DS, Taplin S: **Quality of life of 5-10 year breast cancer survivors diagnosed between age 40 and 49.** *Health Qual Life Outcomes* 2004, 2: 25.

13. Cui Y, Shu XO, Gao YT, Cai H, wen WQ, Ruan ZX, Jin F, Zheng W: **The long-term impact of medical and socio-demographic factors on the quality of life of breast cancer survivors among Chinese women.** *Breast Cancer Res Treat* 2004: 87: 135-147.

14. Diel IJ, Body JJ, Lichinitser MR, Kreuser ED, Dornoff W, Gorbunova VA, Budde M, Bergstrom B: **Improved quality of life after long-term treatment with bisphosphonate ibandronate in patients with metastatic bone disease due to breast cancer.** *Eur J Cancer* 2004, 40: 1704-1712.

15. Dubernard G, Sideris L, Delaloge S, Marsiglia H, Rochard F, Travagli JP, Mathieu MC, Lumbroso J, Spielmann M, Garbay JR, Rouzier R: **Quality of life after sentinel lymph node biopsy in early breast cancer.** *Eur J Surgical Oncol* 2004, 30: 728-734.

16. Efficace F, Biganzoli L, Piccart M, Coens C, van Steen K, Cufer T, Coleman RE, Calvert HA, Gamucci T, Twelves C, Fargeot P, Bottomley A: **Baseline health-related quality of life data as prognostic factors in a phase III multicenter study of women with metastatic breast cancer.** *Eur J Cancer* 2004, 40: 1021-1030.

17. Efficace F, Therasse P, Piccart MJ, Coens C, van Steen K, Welnicka-Jaskiewicz M, Cufer T, Dyczka J, Lichinitser M, Shepherd L, de Haes H, Srangers MA, Bottomley A: **Health-related quality of life parameters as prognostic factors in a nonmetastatic breast cancer population: an international multicenter study.** *J Clin Oncol* 2004, 22: 3381-3388.

18. Engel J, Kerr J, Schlesinger-Raab A, Sauer H, Halzel D. **Quality of life following breast-conserving therapy or mastectomy: results of a 5-year prospective study.** *Breast J* 2004, 10: 223-231.

19. Fallowfield L, Cella D, Cuzick J, Francis S, Locker G, Howll A: **Quality of life of postmenopausal women in the Arimidex, tamoxifen alone or in combination(ATAC) adjuvant breast cancer trial.** *J Clin Oncol* 2004, 22: 4261-4271.

20. Fallowfield LJ: **Evolution of breast cancer treatment: current options and quality-of-life consideration.** *Eur J Oncol Nurs* 2004, 8 (Suppl. 2): S75-82.

21. Flores AM, Hodges LC, Brewer LH. **Recovering shoulder function and quality of life after breast cancer surgery.** *J Sect Women’s Health* 2004, 28: 7-14.

22. Fossati R, Confalonieri C, Mosconi P, Pistotti V, Apolone G: **Quality of life in randomized trials of cytoxic or hormonal treatment of advanced breast cancer.** **Is there added value?** *Breast Cancer Res Treat* 2004, 87: 233-243.

23. Ganz PA, Kwan L, Stanton AL, Krupnick JL, Rowland JH, Meyerowitz BE, Bower JE, Belin TR: **Quality of life at the end of primary treatment of breast cancer: first results from the moving beyond cancer randomized trial.** *J Natl Cancer Inst* 2004, 96: 376-387.

24. Gaston-Johansson F, Lachica EM, Fall-Dickson JM, Kennedy MJ: **Psychological distress, fatigue, burden of care, and quality of life in primary caregivers of patients with breast cancer undergoing autologous bone marrow transplantation.** *Oncol Nurs Forum* 2004, 31: 1161-1169.

25. Giedzinska AS, Meyerowitz BE, Ganz PA, Rowland JH: **Health-related quality of life in a multiethnic sample of breast cancer survivors.** *Ann Behav Med* 2004, 28: 39-51.

26. Goodwin PJ, Ennis M, Bordeleau LJ, Pritchard KT, Trudeau Me, Koo J, Hood N: **Health-related quality of life and psychosocial status in breast cancer prognosis: analysis of multiple variables.** *J Clin Oncol* 2004, 22: 4184-4192.

27. Harwood KV. **Advances in endocrine therapy for breast cancer: considering efficacy, safety, and quality of life.** *J Clin Oncol Nurs* 2004, 8: 629-640.

28. Headley JA, Ownby KK, John LD. **The effect of seated exercise on fatigue and quality of life in women with advanced breast cancer.** *Oncol Nurs Forum* 2004, 31: 977-983.

29. Kershaw T, Northouse L, kritpracha C, Schafenacker A, Mood D: **Coping strategies and quality of life in women with advanced breast cancer and their family caregivers*.*** *Psychol Health* 2004,19: 139-155.

30. Land SR, Kopec JA, Yothers G, Anderson S, Day R, Tang G, Ganz PA, Fisher B, Wolmark N: **Health-related quality of life in axillary node-negative, estrogen receptor-negative breast cancer patients undergoing AC versus CMF chemotherapy: findings from the National Surgical Adjuvant Breast and Bowel Project B-23.** *Breast Cancer Res. Treat* 2004, 86: 153-164.

31. Lee EH, Chun M, Kang S, Lee HJ: **Validation of the functional assessment of cancer therapy-general (FACT-G) scale for measuring the health-related quality of life in Korean women with breast cancer.** *Jap J Clin Oncol* 2004, 34: 393-399.

32. Luoma M, Hakamies-Blomqvist L: **The meaning of quality of life in patients being treated for advanced breast cancer: a qualitative study*.*** *Psycho Oncol* 2004, 13: 729-739.

33. Mustian KM, Katual JA, Gill DL, Roscoe JA, Lang D, Murphy K: **Tai Chi Chuan, health-related quality of life and self-esteem: a randomized trial with breast cancer survivors.** *Support Care Cancer* 2004, 12: 871-876.

34. Oh S, Heflin L, Meyerowitz BE, Desmond KA, Rowland JH, Ganz PA: **Quality of life of breast cancer survivors after a recurrence: a follow-up study.** *Breast Cancer Res Treat* 2004, 87: 45-47.

35. Overcash JA: **Using narrative research to understand the quality of life of older women with breast cancer.** *Oncol Nurs Forum* 2004, 31: 1153-1159.

36. Piao BK, Wang YX, Xie GR, Mansmann U, Matthes H, Beuth J, Lin HS**, Impact of complementary mistletoe extract treatment on quality of life in breast, overian, and non-small cell lung cancer patients.** **A prospective randomized controlled clinical trial.** *Anticancer Res* 2004, 24: 303-309.

37. Rietman J, Dijkstra P, Debreczeni R, geertzen J, Robinson D, de Vries J: **Impairments, disabilities and health related quality of life after treatment of breast cancer: a follow-up study 2.7 years after surgery.** *Disabil Rehabil* 2004, 26: 78-84.

38. Rijnsburger AJ, Essink-Bot ML, van Dooren S, Borsboom GJJM, Seynaeve C, Bartels CCM, Klijn JGM, Tibben A, de Koning HJ: **Impact of screening for breast cancer in high-risk women on health-related quality of life.** *Br J Cancer* 2004, 91: 69-76.

39. Sammarco A: **Enhancing the quality of life of survivors of breast cancer.** *Ann Long Term Care* 2004, 12: 40-45.

40. Sandgren AK, Mullens AB, Erickson SC, Romanek KM, McCal KD: **Confidant and breast cancer patients’ reports of quality of life.** *Qual Life Res* 2004, 13: 155-60.

41. Schreier AM, Williams SA: **Anxiety and quality of life of women receive radiation or chemotherapy for breast cancer.** *Oncol Nurs Forum* 2004, 31: 127-130.

42. Turner J, Hayes S, Reul-Hirche H: **Improving the physical status and quality of life of women treated for breast cancer: a pilot study of a structured exercise intervention.** *J Surg Oncol* 2004, 86: 141-146.

43. Twelves CJ, Miles DW, Hall A: **Quality of life in women with advanced breast cancer treated with docetaxel.** *Clin Breast Cancer* 2004, 5: 216-222.

44. Uzun O, Aslan FE, Selimen D, Koc M: **Quality of life in women with breast cancer in Turkey.** *J Nurs Scholarship* 2004, 36: 207-213.

45. van der Steeg AF, De Vries J, Roukema JA: **Quality of life and health status in breast carcinoma.** *Eur J Surg Oncol* 2004, 30: 1051-1057.

46. Weinfurt KP, Castel LD, Li Y, Timbie JW, Glendenning GA, Schulman KA: **Health-related quality of life among patients with breast cancer receiving zoledronic asid or pamidornate disodium for metastatic bone lesion.** *Med Care* 2004, 42: 164-175

47. Yun YH, Bae SH, Kang IO, Shin KH, Lee R, Kwon SI, Park YS, Lee ES: **Cross-cultural application of the Korean version of the European Organization for Research and Treatment of Cancer (EORTC) Breast-Cancer Speicifc Quality of Life Questionnaire (EORTC QLQ-BR23).** *Support Care Cancer* 2004, 12: 441-445.

**2003**

1. Amichetti M, Caffo O. **Pain after quadrantectomy and radiotherapy for early-stage breast cancer: incidence, characteristics and influence on quality of life. Results from a retrospective study.** *Oncology* 2003, 65: 23-28.

2. Bordeleau L, Szalai JP, Ennis M, Leszcz M, Speca M, Sela R, Doll R, Chochinov HM, Navarro M, Arnold A, Pritchard KI, Bezjak A, Liewellyn-Thomas HA, Sawka CA, Goodwin PJ: **Quality of life in a randomized trial of group psychological support in metastatic breast cancer: overall effects of the intervention and an exploration of missing data.** *J Clin Oncol* 2003, 21: 1944-1951.

3. Brandberg Y, Michelson H, Nilsson B, Bolund C, Erikstein B, Hietanen P, Kaasa S, Nilsson J, Wiklund T, Wilking N, Bergh J: **Quality of life in women with breast cancer during the first year after random assignment to adjuvant treatment with marrow-supported high-dose chemotherapy with cyclophosphamide, thiotepa, and carboplatin or tailored therapy with fluorouracil, epirubicin, and cyclophosphamide: Scandinavian Breast Group Study 9401.** *J Clin Oncol* 2003, 21: 3659-3664

4. Caffo O, Amichetti M, Ferro A, Lucenti A, Valduga F, Galligioni E: **Pain and quality of life after surgery for breast cancer.** *Breast Cancer Res Treat* 2003, 80: 39-48.

5. Carlson LE, Speaca M, Patel KD, Goodey E: **Mindfulness-based stress reduction in relation to quality of life, mood, symptoms of stress and and immune parameters in breast and prostate cancer outpatients.** *Psychosom Med* 2003, 65: 571-581.

6. Chie WC, Chang KJ, Huang CS, Kuo WH: **Quality of life of breast cancer patients in Taiwan: validation of the Taiwan Chinese version of the EORTC QLQ-C30 and EORTC QLQ-BR23.** *Psycho-Oncol* 2003, 12: 729-735.

7. Cocquyt VF, Blondeel PN, Depypere HT, van de Sijpe KA, Daems KK, Monstrey SJ, van Belle SJ: **Better cosmetic results and comparable quality of life skin-sparing mastectomy and immediate autologous breast reconstruction compared to breast conservative treatment.** *Br J Plast Surg* 2003, 56: 462-470.

8. Courneya KS, Mackey JR, Bell GJ, Jones LW, Field CJ, Fairey AS: **Randomized controlled trial of exercise training in postmenopausal breast cancer survivors: cardiopulmonary and quality of life outcomes.** *J Clin Oncol* 2003, 21: 1660-1668.

9. de Haes H, Olschewski M, Kaufmann M, Schumacher M, Jonat W, Sauerbrei W: **Quality of life in goserelin-treated versus cyclophosphamide plus methotrexate plus fluorouracil-treated premenopausal and perimenopausal patients with node-positive early breast cancer: the Zoladex Early Breast Cancer research Association Trialists Group.** *J Clin Oncol* 2003, 21: 4510-4516.

10. de Haes JCJM, Curran D, Aaronson NK, Fentiman IS: **Quality of life in breast cancer patients aged over 70 years, participating in the EORTC 10850 randomized clinical trial.** *Eur J Cancer* 2003, 39: 945-951.

11. Engel J, Kerr J, Schlesinger-Raab A, Sauer H, Holzel D: **Axilla surgery severely affect quality of life: results of a 5-year prospective study in breast cancer patients.** *Breast Cancer Res Treat* 2003, 79: 47-57.

12. Engel J, Kerr J, Schlesinger-Raab A, Sauer H, Holzel D: **Comparison of breast and rectal cancer patients’ quality of life: results of a four year prospective field study.** *Eur J Cancer Care* 2003, 12: 215-223.

13. Engel J, Kerr J, Schlesinger-Raab A, Eckel R, Sauer H, Holzel D: **Predictors of quality of life of breast cancer patients.** *Acta Oncol* 2003, 42: 710-718.

14. Ganz PA, Guadagnoli E, Landdrum MB, Lash TL, Rakowski W, Silliman RA: **Breast cancer in older women: quality of life and psychological adjustment in the 15 months after diagnosis.** *J Clin Oncol* 2003, 21: 4027-4033.

15. Girotto JA, Schreiber J, Nahabedian MY: **Breast reconstruction in the elderly: preserving excellent quality of life.** *Ann Plast Surg* 2003, 50: 572-578.

16. Goodwin PJ, Black JT, Bordeleau LJ, Ganz PA: **Health-related quality-of-life measurment in randomized clinical trials in breast cancer.** **Taking stock.** *J Natl Cancer Inst* 2003, 95: 263-281.

17. Graves KD, Carter CL, Anderson ES, Winett RA: **Quality of life pilot intervention for breast cancer patients: use of social cognitive theory.** *Palliative Supportive Care* 2003, 1: 121-134.

18. Hartl K, Janni W, Kastner R, Sommer H, Strobl B, Rack B, Stauber M**: Impact of medical and demographic factors on long-term quality of life and body image of breast cancer patients.** *Ann Oncol* 2003, 14: 1064-1071.

19. Kerr J, Engel J, Schlesinger-Raab A, Sauer H, Holzel D: **Communication, quality of life and age: results of a 5-year prospective study in breast cancer patients.** *Ann Oncol* 2003, 14: 421-427.

20. Kouloulias V, Matsopoulos G, Kouvaris J, Dardoufas C, Bottomley A, Varela M, Uzunoglu N, Antypas C, Metafa A, Moulopoulos A, Sandilos P, Vlahos L: **Radiotherapy in conjunction with intravenous infusion of 180 mg of disodium pamidronate in management of osteolytic metastases from breast cancer: clinical evaluation, biochemical markers, quality of life, and monitoring of recalcification using assessments of gray-level histogram in plain radiographs.** *Int J Radiat Oncol* *Biol Phys* 2003, 57: 143-157.

21. Luoma ML, Hakamies-Blomqvist L, Sjostrom J, Pluzanska A, Ottoson S, Mouridsen H, Bengtsson NO, Bergh J, Malmstrom P, Valvere V, Tennvall L, Blomqvist C: **Prognostic value of quality of life scores for time to progression (TTP) and overall survival time (OS) in advanced breast cancer.** *Eur J Cancer* 2003, 39: 1370-1376.

22. Mandelblatt J, Figueiredo M, Cullen J: **Outcomes and quality of life following breast cancer treatment in older women: when, why, how much, and what do women want?** *Health Qual life Outcomes* 2003, 1: 45

23. Payne R, Medina E, Hampton JW: **Quality of life concerns in patients with breast cancer: evidence for disparity of outcomes and experiences in pain management and palliative care among African-American women.** *Cancer* 2003, 97 (Suppl. 1): 311-317.

24. Peintinger F, Reitsamer R, Stranzl H, Ralph G: **Comparison of quality of life and arm complaints after axillary lymph node dissection vs. sentinel lymph node biopsy in breast cancer patients.** *Br J Cancer* 2003; 89: 648-652.

25. Radice D, Redaelli A: **Breast cancer management: quality of life and cost considerations.** *Pharmacoeconomics* 2003, 21: 383-396.

26. Rietman JS, Dijkstra PU, Hoekstra HJ, Eisma WH, Szabo BG, Groothoff JW, Geertzen JH: **Late morbidity after treatment of breast cancer in relation to daily activities and quality of life: a systematic review.** *Eur J Surg Oncol* 2003; 29: 229-238.

27. Roemer-Becuwe C, Krakowski I, Conroy T: **Bisphosphonates, pain, and quality of life measurement in randomized clinical trials in breast cancer-Taking stock.** *Bull Cancer* 2003, 90: 1097-1105.

28. Sammarco A: **Quality of life among older survivors of breast cancer.** *Cancer Nurs* 2003, 26: 431-438.

29. Thomas R: **Examining quality of life issues in relation to endocrine therapy for breast cancer.** *Am J Clin Oncol:* *Cancer Clin Trials* 2003, 26 (Suppl. 4): S40-S44.

30. Vacek PM, Winstead-Fry P, Secker-Walker RH, Hooper GJ, Plante DA: **Factors influencing quality of life in breast cancer survivors.** *Qual Life Res* 2003, 12: 527-537.

31. Voogd AC, Ververs JMMA, Vingerhoets AJJM, Roumen RMH, Coebergh JWW, Crommelin MA: **Lymphoedema and reduced shoulder function as indicators of quality of life after axillary lymph node dissection for invasive breast cancer.** *Br J Surg* 2003, 90: 76-81.

**2002**

1. Amir M, Ramati A: **Post-traumatic symptoms, emotional distress and quality of life in long-term survivors of breast cancer: a preliminary research.** *J Anxiety Disord* 2002, 16: 191-206.

2. Beaulac SM, McNair LA, Scott TE, et al: **Lymphedema and quality of life in survivors of early-stage breast cancer.** *Arch Surg* 2002, 137, 1253-1257.

3. Bottomley A, Therasse P: **Quality of life in patients undergoing systematic therapy for advanced breast cancer.** *Lancet Oncol* 2002, 3: 620-628.

4. Cimprich B, Ronis DL, Martinez-Ramos G: **Age at diagnosis and quality of life in breast cancer survivors.** *Cancer Pract* 2002, 10: 85-93.

5. Costantino J: **The impact of hormonal treatments on quality of life of patients with metastatic breast cancer.** *Clinical Ther* 2002, 24 (Suppl. C): C26-C42.

6. Durna EM, Crowe SM, Leader LR, Eden JA: **Quality of life of breast cancer survivors: the impact of hormonal replacement therapy.** *Climacteric* 2002, 5: 266-276.

7. Efficace F, Bottomely A, Collines GS: **Quality of life in breast cancer: measurement issues in breast cancer clinical trials.** *Expert Rev Pharmaeconomic Outcomes Res* 2002, 2: 57-65.

8. Fortner BV, Stepanski EJ, Wang SC, Kasprowicz S, Durrence H: **Sleep and quality of life in breast cancer patients.** *J Pain Symptom Manag* 2002, 24, 471-480.

9. Ganz PA Desmond KA, Leedham B, Rowland JH, Meyerowitz BE, Belin TR: **Quality of life in long-term, disease-free survivors of breast cancer: a follow-up study.** *J Natl Cancer Inst* 2002, 94: 39-49.

10. Genre D, Protiere C, Macquart-Moulin G, Gravis G, Camerlo J, Alzieu C, Maraninchi D, Moatite JP, Viens P: **Quality of life of breast cancer patients receiving high-dose-intensity chemotherapy: impact of length of cycles.** *Support Care Cancer* 2002, 10: 222-230.

11. Gotay CC, Holup JL, Pagano I: **Ethnic differences in quality of life among early breast and prostate cancer survivors.** *Psycho-Oncol* 2002,11: 103-113.

12. Heidemann E, Stoeger H, Souchon R, Hirschmann WD, Bodenstein H, Oberhoff C, Fischer JT, Schulze M, Clemens M, Andreesen R, Mahlke M, Konig M, Scharl A, Fehnle K, Kaufmann M: **Is first-line single-agent mitoxantrone in the treatment of high-risk metastatic breast cancer patients as effective as combination chemotherapy? No difference in survival but higher quality of life were found in a multicenter randomized trial.** *Ann Oncol* 2002, 13: 1717-1729.

13. Kessler TA: **Contextual variables, emotional state, and current and expected quality of life in breast cancer survivors*.*** *Oncol Nurs Forum* 2002, 29: 1109-1116.

14. Kurtz JE, Dufour P: **Strategies for improving quality of life in older patients with metastatic breast cancer.** *Drugs Aging* 2002, 19: 605-622.

15. Kwan W, Jackson J, Weir LM, Dingee C, McGregor G, Olivotto IA: **Chronic arm morbidity after curative breast cancer treatment: prevalence and impact on quality of life.** *J Clin Oncol* 2002, 20: 4242-4248.

16. Michael YL, Berkman LF, Colditz GA, Holmes MD, Kawachi I: **Social networks and health related quality of life in breast cancer survivors: A prospective study.** *J Psychosomatic Res* 2002, 52: 285-293.

17. Modi S, Panageas KS, Duck ET, Bach A, Weinstock N, Dougherty J, Cramer L, Hudis C, Norton L, Seidman A: **Prospective exploratory analysis of the association between tumor response, quality of life, and expenditure among patients receiving paclitaxel monotherapy for refractory metastatic breast cancer.** *J Clin Oncol* 2002, 20: 3665-3673.

18. Mosconi P, Apolone G, Barni S, Secondino S, Sbanotto A, Filliberti A: **Quality of life in breast and colon cancer long-term survivors: an assessment with the EORTC QLQ-C30 and SF-36 questionnaires.** *Tumori* 2002, 88: 110-116.

19. Northouse LL, Mood D, Kershaw T, Schafenacker A, Mellon S, Walker J, Galvin E, Decker V: **Quality of life of women with recurrent breast cancer and their family members.** *J Clin Oncol* 2002, 20: 4050-4064.

20. O’Shaughnessy JA: **Effects of epoetin alfa on cognitive function, mood, asthenia, and quality of life in women with breast cancer undergoing adjuvant chemotherapy.** *Clin Breast Cancer* 2002, 3 (Suppl. 3): S116-S120.

21. Olsson AM, Svensson JH, Sundstrom J, Bergstrom S, Edekling T, Carlsson G, Hansen J, Sevnsson B, Albertsson M: **Erythropoietin treatment in metastatic breast cancer: effects on Hb, quality of life and need for transfusion.** *Acta Oncol* 2002, 41: 517-524.

22. Osoba D, Slamon DJ, Burchmore M, Murphy M: **Effects on quality of life of combined trastuzumab and chemotherapy in women with metastatic breast cancer.** *J Clin Oncol* 2002, 20: 3106-3113.

23. Pandey M, Thomas BC, Ramdas K, Eremenco S, Nair K: **Quality of life in breast cancer patients: validation of FACT-B Malayalam version.** *Qual Life Res* 2002, 11: 87-90.

24. Parker PA, Middleton MS, Kulik JA, **Counterfactual thinking and quality of life among women with silicone breast implants.** *J Behav Med* 2002, 25: 317-335.

25. Pinto BM, Trunzo JJ, Reiss P, Shiu SY: **Exercise participation after diagnosis of breast cancer: trends and effects on mood and quality of life.** *Psycho-Oncol* 2002, 11: 389-400.

26. Shimozuma K, Okamoto T, Katsumata N, Koike M, Tanaka K, Osumi S, Saito M, Shikama N, Watanabe T, Mitsumori M, Yamauchi C, Hisashige A: **Systematic overview of quality of life studies for breast cancer.** *Breast Cancer* 2002, 9: 196-202.

**2001**

1. Amichetti M, Caffo O: **Quality of life in patients with early stage breast carcinoma treated with conservation surgery and radiotherapy. An Italian monoinstitutional study.** *Tumori* 2001, 87: 78-84.

2. Arora NK, Gustafson DH, Hawkins RP, mcTavish F, Cella DF, Pingree S, Mendenhall JH, Mahvi DM**: Impact of surgery and chemotherapy on the quality of life of younger women with breast carcinoma: a prospective study.** *Cancer* 2001, 92: 1288-1298.

3. Carlson LE, Koski T, Gluck S: **Longitudinal effects of high-dose chemotherapy and autologous stem cell transplantation on quality of life in the treatment of metastatic breast cancer.** *Bone Marrow Transpl* 2001, 27: 989-998.

4. Carlsson M, Arman M, Backman M, Hamrin E: **Perceived quality of life and coping for Swedish women with breast cancer who choose complementary medicine.** *Cancer Nurs* 2001, 24: 395-401.

5. Carpenter JS: **The hot flash related daily interference scale: a tool for assessing the impact of hot flashes on quality of life following breast cancer.** *J Pain Symptom Manag* 2001, 22: 979-989.

6. Coates AS, Hurny C, Peterson HF, et al: **Quality of life scores predict outcome in metastatic but not in early breast cancer.** *Breast* 2001, 10 (Suppl. 3): 164-170.

7. Coster S, Poole K, Fallowfield LJ, **The validation of a quality of life scale to assess the impact of arm morbidity in breast cancer patients post-operatively.** *Breast Cancer Res Treat* 2001, 68: 273-282.

8. Day R: **Quality of life and tamoxifen in a breast cancer prevention trial-a summary of findings from the NSABP-1 study.** *Ann Ny Acad Sci* 2001, 949, 143-150.

9. Goodwin PJ: **Economics, quality of life and breast outcomes-Is a balance possible?** *Breast* 2001, 10 (Suppl. 3): 164-170.

10. Heim ME, Kunert S, Ozkan I: **Effects of inpatient rehabilitation on health-related quality of life in breast cancer patients.** *Onkologie* 2001, 24: 268-272.

11. Holzner B, Kemmier G, Kopp M, Moschen R, Schweigkofler H, Dunser M, Margreiter R, Fleischhacker WW, Sperner-Unterweger B: **Quality of life in breast cancer patients. Not enough attention for long-term survivors?** *Psychosomatics* 2001, 42: 117-123.

12. Janni W, Rjosk D, Dimpfl T, Haertl K, Strobl B, Hepp F, Hanke A, Bergauer F, Sommer H: **Quality of life influenced by primary surgical treatment for stage I-III breast cancer-long term follow-up of a matched-pair analysis.** *Ann Surg Oncol* 2001, 8: 542-548.

13. Lee HL, Ku NP, Dow WJ, Pai L: **Factors related to quality of life in breast cancer patients receiving chemotherapy.** *J Nurs Res* 2001, 9: 57-67.

14. Lev EL, Daley KM, Conner NE, Reith M, Fernandez C, Owen SV: **An intervention to increase quality of life and self-care self-efficacy decrease symptoms in breast cancer patients.** *Scholarly Inquiry for Nursing Practice* 2001, 15: 277-294.

15. Lewis JA, Manne SL, DuHamel KN, Vickburg SMJ, Bovjerg DH, Currie V, Winkel G, Redd WH: **Social support, intrusive thoughts, and quality of life in breast cancer survivors.** *J Behav Med* 2001, 24: 231-245.

16. Marsden J, Baum M, A’Hern R, West A, Fallowfield L, Whitehead M, Sacks N: **The impact of hormone replacement therapy on breast cancer patients’ quality of life and sexuality: a pilot study.** *Br J Menopause Sco* 2001, 7: 85-87.

17. McInnes JA, Konbf MT: **Weight gain and quality of life in women treated with adjuvant chemotherapy for early stage breast cancer.** *Oncol Nurs Forun* 2001, 28: 675-696.

18. Mihailova Z, Butorin N, Antonov R, Toporov N, Popova V: **Evaluation of the Bulgarian version of the European Organization for Research and Treatment of Cancer quality of life questionnaire C30 (version 2) and breast cancer module (BR23) on the psychometric properties of breast cancer patients under adjuvant chemotherapy. Prognostic value of estrogen and progesterone receptors to quality of life.** *J Balkan Union of Oncol* 2001, 6: 415-424.

19. Molenaar S, Sprangers MAG, Rutgers EJT, Luiten EJT, Mulder J, Boss MM, van Everdingen JJE, Oosterveld P, de Haes HCJM: **Decision support for patients with early-stage breast cancer: effects of an interactive breast cancer CDROM on treatment decision, satisfaction, and quality of life.** *J Clin Oncol* 2001, 19: 1676-1687.

20. Mosconi P, Colozza M, Laurentiis MD, Placido SD, Maltoni M: **Survival, quality of life and breast cancer.** *Ann Oncol* 2001, 12 (Suppl 3): S15-S19.

21. Nagel GC, Schmidt S, Strauss BM, Katenkamp D: **Quality of life in breast cancer patients: a cluster analytic approach.** *Breast Cancer Res Treat* 2001, 68: 75-87.

22. Nissen MJ, Swenson KK, Ritz LJ, Farrell JB, Sladek ML, Lally RM. **Quality of life after breast carcinoma surgery: a comparison of three surgical procedures.** *Cancer* 2001, 91:1238-1246.

23. Partridge AH, Bunnell CA, Winer EP: **Quality of life issues among women undergoing high-dose chemotherapy for breast cancer.** *Breast Dis* 2001, 14: 41-50.

24. Perez DJ, Williams SM, Christensen EA, McGee RO, Camplbell AV**: A longitudinal study of health related quality of life and utility measures in patient with advanced breast cancer.** *Qual Life Res* 2001, 10: 578-593.

25. Sammarco A: **Perceived social support, uncertainty, and quality of life of younger breast cancer survivors.** *Cancer Nurs* 2001, 24: 212-219.

26. Sammarco A: **Psychological stages and quality of life of women with breast cancer.** *Cancer Nurs* 2001, 24: 272-277.

27. Shapiro SL, Lopez AM, Schwartz GE, Bootzin R, Figueredo AJ, Braden C, Kurker SF: **Quality of life and breast cancer: relationship to psychological variables.** *J Clin Psychol* 2001, 57: 501-509.

**2000**

1. Bower JE, Ganz PA, Desmond KA, Rowland JH, Meyetowitz BE, Belin TR: **Fatigue in breast cancer survivors: occurrence, correlates, and impact on quality of life.** *J Clin Oncol* 2000, 18: 743-753.

2. Braden CJ, Mishel MH: **Highlights of the self-help intervention project (SHIP): health-related quality of life during breast cancer treatment.** *Innovations Breast Cancer Care* 2000, 5: 27-34.

3. Brandberg Y, Malm M, Blomqvist L: **A prospective and randomized study (SVEA): comparing effects of three methods for delayed breast reconstruction on quality of life. Patient-defined problem areas of life and cosmetic result.** *Plast Reconstr Surg* 2000, 105: 66-74.

4. Broeckel JA, Jacobsen PB, Balducci L, Horton J, Lyman GH: **Quality of life after adjuvant chemotherapy for breast cancer.** *Breast Cancer Res Treat* 2000, 62: 141-150.

5. Coates AS, Hurny C, Peterson HF, Bernhard J, Castinglione-Gertsch M, Gelberg D, Goldhirsch A: **Quality of life scores predict outcome in metastatic but not early breast cancer. International Breast Cancer Study Group.** *J Clin Oncol* 2000, 18: 3768-3774.

6. Cousson-Gelie F: **Breast cancer, coping and quality of life: a semi-prospective study.** *Eur Rev Applied Psy* 2000, 50: 315-320.

7. Curran D, Aaronson N, Standaert B, Molenberghs G, Therasse P, Ramirez A, Koopmanschap M, Eder H, Piccart, M: **Summary measures and statistics in the analysis of quality of life data: an example from an EORTC-NCIC-SAKK locally advanced breast cancer study.** *Eur J Cancer* 2000, 36: 834-844.

8. Dahlbender RW, Maiterth C, Meder G, Klaus W, Kreienberg R, Kuchn T: **Surgery strategies, quality of life, and conspicuous psychosocial constellations in women with breast cancer.** *Breast Cancer Res Treat* 2000, 64: 559-…..[treatment-surgery]

9. Dow KH, Lafferty P: **Quality of life, survivorship, and psychosocial adjustment of young women with breast cancer after breast conserving surgery and radiation therapy.** *Oncol Nurs Forum* 2000, 27: 1555-1564.

10. Ganz PA: **Quality of life across the continuum of breast cancer care.** *Breast J* 2000, 6: 324-330.

11. Hakamies-Blomqvist L, Luoma ML, Sjostrom J, Pluzanska A, Sjodin M, Mouridsen H, Ostenstad B, Mjaaland I, Ottosson-Lonn S, Bergh J, Malmstrom P, Blomqvist C: **Quality of life in patients with metastatic breast cancer receiving either docetaxel or sequential methotrexate and 5-fluorouracil. A multicenter randomized phase III trial by the Scandinavian Breast Group.** *Eur J Cancer* 2000, 36: 1411-1417.

12. Jansen SJT, Stiggelbout AM, Nooij MA, Noordijk EM, Kievit J: **Response shift in quality of life measurement in early-stage breast cancer patients undergoing radiotherapy.** *Qual Life Res* 2000, 9: 603-615.

13. Joly F, Espie M, Marty M, Heron JF, Henry-Amar M: **Long-term quality of life in premenopausal women with node-negative localized breast cancer treated with or without adjuvant chemotherapy.** *Br J Cancer* 2000, 83: 577-582.

14. Kenny P, King MT, Sheill A, Seymour J, Hall J, Langlsnds A, Boyages J: **Early stage breast cancer, costs and quality of life one year after treatment by mastectomy or conservative surgery and radiation therapy*.*** *Breast* 2000; 9: 37-44.

15. King MT, Kenny P, Shiell A, Hall J, Boyages J: **Quality of life three months and one year after first treatment for early stage breast cancer: influence of treatment and patient characteristics.** *Qual Life Res* 2000, 9: 789-800.

16. Kramer JA, Curran D, Piccart M, de Haes JC, Bruning PF, Klijn JG, Bontenbal M, van Pottelsberghe C, Groenvold M, Paridaens R: **Randomized trial of paclitaxel versus doxorubicin as first-line chemotherapy for advanced breast cancer: quality of life evaluation using the EORTC QLQ-C30 and the Rotterdam Symptom Checklist.** *Eur J Cancer* 2000, 36: 1488-1497.

17. Kramer JA, Curran D, Piccart M, de Haes JC, Bruning PF, Klijn JG, van Hoorebeeck I, Paridaens R: **Identification and interpretation of clinical and quality of life prognostic factors for survival and response to treatment in firs-line chemotherapy in advanced breast cancer.** *Eur J Cancer* 2000, 36: 1498-1506.

18. Kuehn T, Klauss W, Darsow M, Regele S, Flock F, Maiterth C, Dahlbender R, Wendt I, Kreienberg R: **Long term morbidity following axillary dissection in breast cancer patients: clinical assessment, significance for life quality and the impact of demographic, oncologic and therapeutic factors.** *Breast Cancer Res Treat* 2000, 64: 275-286.

19. Macquart-Moulin G, Viens P, Palangie T, Bouscary ML, Delozier T, Roche H, Janvier M, Fabbro M, Moatti JP: **High-dose sequential chemotherapy with recombination granulocyte colony-stimulating factor and repeated stem-cell support for inflammatory breast cancer patients: does impact on quality of life jeopardize feasibility and acceptability of treatment?** *J Clin Oncol* 2000, 18: 754-764.

20. Montazeri A, Harirchi I, Vahdani M, Khaleghi F, Jarvandi S, Ebrahimi M, Haji-Mahmoodi M: **The EORTC breast cancer-specific quality of life questionnaire (EORTC QLQ-BR23): translation and validation study of the Iranian version.** *Qual Life Res* 2000, 9: 177-184.

21. Osoba D: **Health related quality of life assessment in breast and prostate cancer.** *J Clin Outcomes Manage* 2000, 7: 53-66.

22. Pandey M, Sebastian P, Ahamed IM, Ramdas K, Thomas BC, Nair MK: **A case-control study into the quality of life of women with breast cancer.** *Cancer Strategy* 2000, 2: 61-68.

23. Pandey M, Singh SP, Behere PB, Roy SK, Singh S, Shukla VK: **Quality of life in patients with early and advanced carcinoma of breast.** *Eur J Surg Oncol* 2000, 26: 20-24.

24. Riccardi A, Tinelli C, Brugnatelli S, Pugliese P, Giardina V, Giordano M, Danova M, Richetti A, Fava S, Rinaldi E, Fregoni V, Trotti G, Poli A: **Doubling of the epirubicin dosage within the 5-fluorouracil, epirubicin and cyclophosphamide regimen: a prospective randomized, multicentric study on antitumor effect and quality of life in advanced breast cancer.** *Int J Oncol* 2000, 16: 769-776.

25. Ritz LJ, Nissen MJ, Swenson KK, Farrell JB, Sperduto PW, Sladek ML, Lally RM, Schroeder LM: **Effects of advanced nursing care on quality of life and cost outcomes in women diagnosed with breast cancer.** *Oncol Nurs Forum* 2000, 27: 923-932.

26. Rustoen T, Begnum S: **Quality of life in women with breast cancer-a review of the literature and implications for nursing practice.** *Cancer Nurs* 2000, 23: 416-421.

27. Shimozuma K, Sonoo H, Ichihara K, Tanaka K: **The prognostic value of quality of life scores: preliminary results of an analysis of patients with breast cancer*.*** *Surg Today* 2000, 30: 255-261.

28. Stein KD, Jacobsen PB, Hann DM, Greenberg H, Lyman G: **Impact of hot flashes on quality of life among postmenopausal women being treated for breast cancer.** *J Pain Symptom Manage* 2000, 19: 436-445.

29. Wengstrom Y, Haggmark C, Strander H, Forsberg C: **Perceived symptoms and quality of life in women with breast cancer receiving radiation therapy.** *Eur J Oncol Nurs* 2000, 4: 78-90.

30. Whelan TJ, Levine M, Julian J, Kirkbride P, Skingley P: **The effects of radiation therapy on quality of life of women with breast carcinoma: results of a randomized trial. Ontario Clinical Oncology Group.** *Cancer* 2000, 88: 2260-2266.

**1999**

1. Amichetti M, Caffo O, Arcicasa M, Roncadin M, Lora O, Rigon A, Zini G, Armaroli L, Coghetto F, Zorat P, Neri S, Teodorani N: **Quality of life in patients with ductal carcionoma in situ of the breast treated with conservative surgery and postoperative irradiation.** *Breast Cancer Res Treat* 1999, 54: 109-115.

2. Andersen MR, Urban N: **Involvement in decision-making and breast cancer survivors quality of life.** *Ann Behave Med* 1999, 21: 201-209.

3. Ashing-Giwa K, Ganz PA, Petersen L: **Quality of life of African-American and white long term breast carcinoma survivors.** *Cancer* 1999, 85: 418-426.

4. Ashing-Giwa K: **Quality of life and psychological outcome in long-term survivors of breast cancer: a focus on African-American women.** *J Psychosoc Oncol* 1999, 17: 47-62.

5. Bernhard J, Castiglione-Gertsch M, Schmitz SFH, Castiglione-Gertsch M, Cavalli F, Morant R, fey MF, Bonnefoi H, Goldhirsch A, Hurny C: **Quality of life in postmenopausal patients with breast cancer after failure of tamoxifen: formestane versus megestrol acetate as second-line hormonal treatment. Swiss Group for Clinical Cancer Research (SAKK).** *Eur J Cancer* 1999, 35: 913-920.

6. Bull AA, Meyerowitz BE, Hart S, Mosconi P, Apolone G, Liberati A: **Quality of life in women with recurrent breast cancer.** *Breast Cancer Res Treat* 1999, 54: 47-57.

7. Chie WC, Huang CS, Chen JH, Chang KJ: **Measurement of the quality of life during different clinical phase of breast cancer.** *J Formosan Med Assoc* 1999, 98: 254-260.

8. Cotton SP, Levine EG, Fitzpatrick CM, Dold KH, Targ E: **Exploring the relationship among spiritual well-being, quality of life, and psychological adjustment in women with breast cancer.** *Psycho-Oncol* 1999, 8: 429-438.

9. Day R, Ganz PA, Costantino JP, Cronin WM, Wickerham DL, Fisher B: **Health-related quality of life and tamoxifen in breast cancer prevention: a report from the National Surgical Adjuvant Breast and Bowel Project P-1 Study.** *J Clin Oncol* 1999, 17: 2659-2669.

10. Dow KH. Options and decisions: **Exploring quality of life issues in recurrent breast cancer.** *Innovations Breast Cancer* *Care* 4: 39-44.

11. Fairclough DL, Fetting JH, Cella D, Wonson W, Moinpour CM: **Quality of life and quality adjusted survival for breast cancer patients receiving adjuvant therapy. Eastern Cooperative Oncology Group (ECOG).** *Qual Life Res* 1999, 8: 723-731.

12. Followfield LJ, Leaity SK, Howell A, Benson S, Cella D: **Assessment of quality of life in women undergoing hormonal therapy for breast cancer: validation of an endocrine symptom subscale for the FACT-B.** *Breast Cancer Res Treat* 1999, 55: 189-199.

13. Hann DM, Garovoy N, Finkelstein B, Jacobsen PB, Azzarello LM, Fields KK: **Fatigue and quality of life in breast cancer patients undergoing autologous stem cell transplantation: a longitudinal comparative study.** *J Pain Symptom Manage* 1999, 17: 313-319.

14. Klotz LK, Lakomy JM, Deardorff KU: **Impact of complementary healing modalities on quality of life and treatment adherence for a maily with breast cancer: a case study approach.** *Int J Hum Caring* 1999, 3: 7-13.

15. Lachaine J, Laurier C, Langleben A, Vaillant L: **Cost-effectiveness and quality of life evaluation of ondansetron and metoclopramide for moderately emetogenic chemotherapy regimens in breast cancer.** *Crit Rev Oncol Hematol* 1999, 32: 105-112.

16. Leedham B, Ganz PA: **Psychological concerns and quality of life in breast cancer survivors.** *Cancer Invest* 1999, 17: 342-348.

17. Longman AJ, Braden CJ, Mishel MH: **Side-effects burden, psychological adjustment, and life quality in women with breast cancer: pattern of association over time.** *Oncol Nurs Forum* 1999, 26: 909-915.

18. Macquart-Moulin G, Viens P, Genre D, Bouscary ML, Resbeut M, Gravis G, Camerlo J, Maraninchi D, Moatti JP: **Concomitant chemotherapy for patients with nonmetastatic breast carcinoma: side effects, quality of life, and organization.** *Cancer* 1999, 85: 2190-2199.

19. McLachlan SA, Pintillie M, Tannock IF: **Third line chemotherapy in patients with metastatic breast cancer : an evaluation of quality of life and cost.** *Breast Cancer Res Treat* 1999, 54: 213-223.

20. McMasters KM, Hunt KK: **Neoadjuvant chemotherapy, locally advanced breast cancer, and quality of life.** *J Clin Oncol* 1999, 17: 441-444.

21. Northouse LL, Caffey M, Deichelbohrer L, Schmidt L, Guziatek-Trojniak L, West S, Kershaw T, Mood D: **The quality of life of African women with breast cancer.** *Res Nurs Health* 1999, 22: 449-460.

22. Okuyama T, Korenaga D, Tamura S, Maekawa S, Kurose S, Ikeda T, Sugimachi K: **Quality of life following surgery for vertebral metastases from breast cancer.** *J Surg Oncol* 1999, 70: 60-63.

23. Osoba D, Burchmore M: **Health-related quality of life in women with metastatic breast cancer treated with trastuzumab (Herceptin).** *Semin Oncol* 1999, 26 (Suppl. 12): 84-88.

24. Pusic A, Thompson TA, Kerrigan CL, Sargeant R, Slezak S, Chang BW, Kelzisouer KJ, Manson PN: **Surgical options for early-stage breast cancer: factors associated with patient choice and postoperative quality of life.** *Plast Reconstr Surg* 1999, 104: 1325-1333.

25. Shimozuma K, Ganz PA, Petersen L, Hirji K: **Quality of life in the first year after breast cancer surgery: rehabilitation needs and patterns of recovery.** *Breast Cancer Res Treat* 1999, 56: 45-57.

26. Velanovich V, Szymanski W: **Quality of life of breast cancer patients with lymphedema: high-dose versus standard-dose chemotherapy.** *Am J Surg* 1999, 177: 184-187.

27. Wapnir IL, Cody RP, Greco RS: **Subtle differences in quality of life after breast cancer surgery.** *Ann Surg Oncol* 1999, 6: 359-366.

28. Wengstrom Y, Haggmark C, Strander H, Forsberg C: **Effects of a nursing intervention on subjective distress, side-effects and quality of life of breast cancer patients receiving curative radiation therapy: a randomized study.** *Acta Oncol* 1999, 38: 763-770.

29. Wenzel LB, Fairclough DL, Brady MJ, Cella D, Garrett KM, Kluhsman BC, Crane LA, Marcus AC: **Age-related differences in the quality of life of breast carcinoma patients after treatment.** *Cancer* 1999, 86: 1768-1774.

30. Westman G, Ahlgren J, Jansson T, Bergh J: **Cross-comparison of two quality of life instruments used in a randomized study of combination chemotherapy in advanced breast cancer.** *Acta Oncol* 1999; 38: 567-572.

**1998**

1. Ashbury FD, Cameron C, Mercer SL, Fitch M, Nielsen E: **One-on-one peer support and quality of life for breast cancer patients.** *Patient Educ Couns* 1998, 35: 89-100.

2. Bernhard J, Hurny C, Coates AS, Peterson HF, Castiglione-Gertsch M, Gelber RD, Galligioni E, Marini G, Thurlimann B, Forbes JF, Goldhirsch A, Senn HJ, Rudenstam CM: **Factors affecting baseline quality of life in two international adjuvant breast cancer trials. International Breast Cancer Study Group (IBCSG).** *Br J Cancer* 1998, 78: 686-693.

3. Bernhard J, Peterson HF, Coates AS, Gusset H, Isley M, Hinkle R, Gelber RD, Castiglione-Gertsch M, Hurny C: **Quality of life of assessment in International Breast Cancer Study Group (IBCSG) trials: practical issues and factors associated with missing data.** *Stat Med* 1998, 17: 587-601.

4. Bloom JR, Stewart SL, Johnston M, Banks P: **Intrusiveness of illness and quality of life in young women with breast cancer.** *Psycho-Oncol* 1998, 7: 89-100.

5. Carlson RW: **Quality of life issues in the treatment of metastatic breast cancer.** *Oncology* 1998, 12 (Suppl. 4): 27-31.

6. Carpenter JS, Andrykowski MA, Cordova M, Cunningham L, Studts J, McGrath P, Kenady D, Sloan D, Munn R: **Hot flashes in postmenopausal women treated for breast carcinoma: prevalence, severity, correlates, management, and relation to quality of life.** *Cancer* 1998, 82: 1682-1691.

7. Coates A, Gebski V: **Quality of life studies of the Australian New Zealand Breast Cancer Trials Group: approaches to missing data.** *Stat Med* 1998, 17: 5330540.

8. Curran D, van Dongen JP, Aaronson NK, Kiebert G, Fentiman IS, Mignolet F, Bartelink H: **Quality of life of early-stage breast cancer patients treated with radical mastectomy or breast conserving procedures: results of EORTC trial 10801.** *Eur J Cancer* 1998, 34; 307-314.

9. de Haes JC, Olschewski M: **Quality of life assessment in a cross cultural context: use of the Rotterdam Symptom Checklist in a multinational randomized trial comparing CMF and Zoladex (Goserlin) treatment in early breast cancer.** *Ann Oncol* 1998, 9: 745-750.

10. Dorval M, Maunsell E, Deschenes L, Brisson J, Masse B: **Long-term quality of life after breast cancer: comparison of 8-year survivors with population controls.** *J Clin Oncol* 1998, 16: 487-494.

11. Dorval M, Maunsell E, Deschenes L, Brisson J: **Type of mastectomy and quality of life for long term breast carcinoma survivors.** *Cancer* 1998, 83: 2130-2138.

12. Ferrell BR, Grant M, Funk B, Otis-Green S, Garcia N: **Quality of life in breast cancer. Part II: psychological and spiritual well-being.** *Cancer Nurs* 1998, 21: 1-9.

13. Ferrell BR, Grant M, Funk B, Otis-Green S, Garcia N: **Quality of life in breast cancer survivors: implications for developing support services.** *Oncol Nurs Forum* 1998, 25: 887-895.

14. Ganz PA, Day R, Costantino J: **Compliance with quality of life data collection in the National Surgical Adjuvant Breast Cancer and Bowel Project (NSABP) Breast Cancer Prevention Trial.** *Stat Med* 1998, 17: 613-622.

15. Ganz PA, Rowland JH, Desmond K, Meyerowitz BE, Wyatt GE: **Life after breast cancer: understanding women’s health-related quality of life and sexual functioning.** *J Clin Oncol* 1998, 16: 501-514.

16. Ganz PA, Rowland JH, Meyerowitz BE, Desmond KA: **Impact of different adjuvant therapy strategies on quality of life in breast cancer survivors.** *Recent Results Cancer Res* 1998, 152: 396-411.

17. Gelber RD, Bonetti M, Cole BF, Gelber S, Goldhirsch A: **Quality of life assessment in the adjuvant setting: is it relevant? International Breast Cancer Study Group.** *Recent Results Cancer Res* 1998, 152: 373-389.

18. Hann DM, Jacobson P, Martin S, et al: **Fatigue and quality of life following radiotherapy for breast cancer: a comparative study.** *J Clin Psychol Med* *S* 1998, 5: 19-33.

19. Hurny C, Bernhard J, Coates A: **Quality of life in the International Breast Cancer Study Group: past, present, future.** *Recent Results Cancer* Res 1998, 152: 390-395.

20. Kissane DW, Clarke DM, Ikin J, Bloch S, smith GC, Vietta L, McKenzie DP: **Psychological morbidity and quality of life in Australian women with early-stage breast cancer: a cross-sectional survey.** *Med J Australia* 1998, 169: 192-196.

21. Kurihara T, Higashi Y, Suemasu K, Tabei T, Ishiguro S, Iino Y, Morishita Y, Takeda F: **Multidrug-resistant recurrent breast cancer which responded to medroxyprogesterone acetate showing a remarkable improvement in the quality of life: report of a case and the role of team medical care.** *Surg Today* 1998, 28: 979-984.

22. Lindley C, Vasa S, Sawyer WT, Winer EP: **Quality of life and preferences for treatment following systematic adjuvant therapy for early-stage breast cancer.** J *Clin Oncol* 1998, 16: 1380-1387.

23. McLachlan SA, Devins GM, Goodwin PJ: **Validation of the European Organization for Research and Treatment of Cancer Quality of Life questionnaire (QLQ-C30) as a measure of psychosocial function in breast cancer patients.** *Eur J Cancer* 1998, 34: 510-517.

24. Ozyilkan O, Baltali E, Tekuzman G, Firat D: **The impact of diagnosis and treatment on the quality of life in breast cancer patients.** *Neoplasma* 1998, 45: 50-52.

25. Rummans TA, Frost M, Suman VJ, Taylor M, Novotny P, Gendron T, Johnson R, Hartmann L, Dose AM, Evans RW: **Quality of life and pain in patients with recurrent breast and gynecologic cancer.** *Psychosomatics* 1998, 39: 437-445.

**1997**

1. Bernhard J, Hurny C, Coates AS, Peterson HF, Castiglione-Gertsch M, Gelber R, Goldhirsch A, Senn HJ, Rudenstam CM: **Quality of life assessment in patients receiving adjuvant therapy for breast cancer: the IBCSG approach. The International Breast Cancer Study Group.**  *Ann Oncol* 1997, 8: 825-835.

2. Brady MJ, Cella DF, Mo F, Bonomi AE, Tulsky DS, Lloyd SR, Deasy S, Cobleigh M, Shiomoto G: **Reliability and validity of the Functional Assessment of Cancer Therapy Breast Quality of Life instrument.** *J Clin Oncol* 1997, 15: 974-986.

3. Couneya KS, Friedenreich CM: **Relationship between exercise during treatment and current quality of life among survivors of breast cancer.** *J Psychosoc Oncol* 1997, 15: 35-57.

4. El-Sharkawi FM, Sakr MF, Atta HY, Ghanem HM: **Effect of different modalities of treatment on the quality of life of breast cancer patients in Egypt.** *East Mediterranean Health J* 1997, 3: 68-81.

5. Ferrell BR, Grant M, Garcia N: **Quality of life in breast cancer. Part I: physical and social well-being.** *Cancer Nurs* 1997, 20: 398-408.

6. Ferrell BR, Grant MM, Funk B, Otis-Green S, Garcia N: **Quality of life in breast cancer survivors as identified by focus groups.** *Psycho-Oncol* 1997, 6: 13-23.

7. Griffiths A, Beaver K. Pilot study reports: **Quality of life during high dose chemotherapy for breast cancer.** *Int J Palliat Nurs* 1997, 3: 138-144.

8. Hann DM, Jacobsen PB, Martin SC, Kronish LE, Azzarello LM, Fields KK: **Quality of life following bone marrow transplantation for breast cancer: a comparative study.** *Bone Marrow Transplant* 1997, 19: 257-264.

9. Hart S, Meyerowitz BE, Apolone G, Mosconi P, Liberati A: **Quality of life among mastectomy patients using external breast prostheses.** *Tumori* 1997, 83: 581-586.

10. Irwig L, Bennetts A: **Quality of life after breast conservation or mastectomy: a systematic review.** *Aust N Z J Surg* 1997, 67: 750-754.

11. Knapp J: **Sexual function as a quality of life issue: the impact of breast cancer treatment.** *J Gynecol Oncol Nurs* 1997, 7: 37-40.

12. Lee CO: **Quality of life and breast cancer survivors: psychological and treatment issues.** *Cancer Pract* 1997, 5: 309-316.

13. Makar K, Cumming CE, Lees AW, Hundleby M, Nabholtz J, Kieren DK, Jenkins H, Wentzel C, Handman M, Cumming DC: **Sexuality, body image, and quality of life after high dose or conventional chemotherapy for metastatic breast cancer.** *Canadian J Human Sexuality* 1997, 6: 1-8.

14. Street RL, Voigt B: **Patient participation in deciding breast cancer treatment and subsequent quality of life.** *Med Decis Making* 1997, 17: 298-306.

15. Tate DG, Riley BB, Perna R, Roller S: **Quality of life issues among women with physical disabilities or breast cancer.** *Arch Phys Med Rehabil* 1997, 78 (Suppl. 5): S18-25.

16. Weitzner MA, Meyers CA, Stuebing KK, Saleeba AK: **Relationship between quality of life and mood in long-term survivors of breast cancer treated with mastectomy.** *Support Care Cancer* 1997, 5: 241-248.

**1996**

1. Andrykowski MA, Curran SL, Studts JL, Cunningham L, Carpenter JS, McGrath PC, Sloan DA, Kenady DE: **Psychological adjustment and quality of life in women with breast cancer and benign breast problems-a controlled comparison.** *J Clin Epidemiol* 1996, 49: 827-834.

2. Bonneterre J, Schraub S, Lecomte S, Mercier M: **Quality of life as an outcome in breast cancer. Clinical application*.*** *Pharmacoeconomics* 1996, 9 (Suppl. 2): 23-29.

3. Buzdar AU, Hortobagyi GN, Frye D, et al: **Second-line chemotherapy for metastatic breast cancer including quality of life issues.** *Breast* 1996, 5: 312-317.

4. Carlsson M, Hamrin E: **Measurement of quality of life in women with breast cancer. Development of a life satisfaction questionnaire (LSQ-32) and a comparison with the EORTC-C30.**  *Qual Life Res* 1996, 5: 265-274.

5. Dow KH, Ferrell BR, Leigh S, Ly J, Gulasekaram P: **An evaluation of the quality of life among long-term survivors of breast cancer.** *Breast Cancer Res Treat* 1996, 39: 261-273.

6. Ferrell BR, Grant M, Funk B, Garcia N, Otis-Green S, Schaffner MLJ: **Quality of life in breast cancer.** *Cancer Pract* 1996, 4: 331-340.

7. Ganz PA, Coscarelli A, Fred C, Kahn B, Polinsky ML, Petersen L: **Breast cancer survivors: psychological concerns and quality of life.** *Breast Cancer Res Treat* 1996, 38: 183-199.

8. Hietanen PS: **Measurement and practical aspects of quality of life in breast cancer.** *Acta Oncol* 1996, 35: 39-42.

9. Hurny C, Bernhard J, Coates AS, Castiglione-Gertsch M, Peterson HF, Gelber RD, Forbes JF, Rudenstam CM, Simoncini E, Crivellari D, Goldhirsch A, Senn HJ: **Impact of adjuvant therapy on quality of life in women with node-positive operable breast cancer. International Breast Cancer Study Group.** *Lancet* 1996, 347: 1279-1284.

10. Larsen J, Gradulf A, Nordstrom G, Bjorkstrand B, Ljungman P: **Health-related quality of life in women with breast cancer undergoing atologous stem-cell transplantation.** *Cancer Nurs* 1996,19: 368-375.

11. Marchioro G, Azzarello G, Checchin F, Perale M, Segati R, Sampognaro E, Rosetti F, Franchin A, Pappagallo GL, Vinate O: **The impact of a psychological intervention on quality of life in non-metastatic breast cancer.** *Eur J Cancer* 1996, 32: 1612-1615.

12. Maunsell E, Brisson J, Deschenes L, Frasure-Smith N: **Randomized trial of a psychologic distress screening program after breast cancer: effects on quality of life.**  *J Clin Oncol* 1996, 14: 2747-2755.

13. McQuellon RP, Craven B, Russell GB, Hoffman S, Cruz JM, Perry JJ, Hurd DD: **Quality of life in breast cancer patients before and after autologous bone marrow transplantation.** *Bone Marrow Transplant* 1996, 18: 579-584.

14. Sprangers MAG, Groenvold M, Arraras JI, Franklin J, te Velde A, Muller M, Franzini L, Williams A, de Haes HC, Hopwood P, Cull A, Aaronson NK: **The European Organization for Research and Treatment of Cancer breast cancer-specific quality-of-life questionnaire module: first results from a three-country field study.** *J Clin Oncol* 1996, 14: 2756-2768.

15. Swain SM, Rowland J, Weinfurt K, Berg C, Lippman ME, Walton L, Egan E, King D, Spertus I, Honig SF: **Intensive outpatient adjuvant therapy for breast cancer: results of dose escalation and quality of life**. *J Clin Oncol* 1996, 14: 1565-1572.

**1995**

1. Berressem P, Frech S, Hartleb M: **Additional therapy with polyerga improve immune reactivity and quality of life in breast cancer patients during rehabilitation.** *Tumordiagn Ther* 1995, 16: 45-48.

2. Bertsch LA, Donaldson G: **Quality of life analysis from vinorelbine (Navelbine) clinical trials of women with metastatic breast cancer.** *Semin Oncol* 1995, 22 (Suppl. 5): 45-54.

3. Fallowfield LJ: **Assessment of quality of life in breast cancer*.*** *Acta Oncol* 1995, 34: 689-694.

4. Ganz PA Day R, Ware JE, Redmond C, Fisher B: **Baseline quality-of-life assessment in the National Surgical Adjuvant Breast and Bowel Project Breast Cancer Prevention Trial.** *J Natl Cancer Inst* 1995, 87, 1372-1382.

5. Liberati A: **The GIVIO trial on the impact of follow-up care on survival and quality of life in breast cancer patients.** **Interdisciplinary Group for Cancer Care Evaluation.**  *Ann Oncol* 1995, 6 (Suppl. 2): 41-46.

6. Osoba D: **Health-related quality of life as a treatment endpoint in metastatic breast cancer.** *Can J Oncol* 1995, 5 (Suppl. 1): 47-53.

7. Seidman AD, Portenoy R, Yao TJ, Lepore J, Mont EK, Kortmansky J, Onetto N, Ren L, Grechko J, Beltangady M, et al: **Quality of life in phase II trials-A study of methodology and predictive value in patients with advanced breast cancer treated with paclitaxel, plus granulocyte colony stimulating factor.** *J Natl Cancer Inst* 1995, 187: 1316-1322.

8. Shimozuma K, Sonoo H, Ichihara K, Miyake K, Kurebayashi J, Ota K, Kiyono T: **The impact of breast conserving treatment and mastectomy on the quality of life in early-stage breast cancer patients.** *Breast Cancer* 1995, 2: 35-43.

9. Shimozuma K, Sonoo H, Ichihara K: **Medical economics and quality of life: analysis of factors the influence the perception of medical cost by post-surgical breast cancer patients.** *Breast Cancer* 1995’ 2: 143-153.

10. Shimozuma K, Sonoo H, Ichihara K: **Analysis of the factors influencing the quality of life of patients with advanced or recurrent breast cancer.** *Surg Today* 1995, 25: 874-882.

**1994**

1. Busch P, Schwendener P, Leu RE, von Dach B, Castiglino M: **Life quality assessment of breast cancer patients receiving adjuvant therapy using incomplete data.** *Health Econ* 1994, 3: 213-220.

2. Fallowfield L: **Quality of life in the elderly women with breast cancer treated with tamoxifen and surgery or tamoxifen alone.** *J Women’s Health* 1994, 3: 17-20.

3. Ferrans CE: **Quality of life through the eyes of survivors of breast cancer.** *Oncol Nurs Forum* 1994, 21: 1645-1651.

4. Ferrero J, Brisson J, Deschenes L, et al: **Mental adjustment to cancer and quality of life in breast cancer patients-An exploratory study.** *Psycho-Oncol* 1994, 3: 223-232.

5. Fox CM, Harper AP, Hyner GC, Lyle RM: **Loneliness, emotional repression, marital quality, and major life events in women who develop breast cancer*.*** *J Community Health* 1994, 19: 476-482.

6. Ganz PA: **Breast cancer in older women: quality of life considerations.** *Cancer Conrol* 1994, 1: 372-379.

7. Ghezzi P, Magnanini S, Rinaldini M, et al: **Impact of follow-up testing on survival and health related quality of life in breast cancer patients-A multicenter randomized controlled trial.** *JAMA* 1994, 271: 1587-1592.

8. Graydon JE: **Women with breast cancer: their quality of life following a course of radiation therapy.** *J Adv Nurs* 1994, 19: 617-622.

9. Hurny C, Bernhard J, Coates A, Castiglione M, Peterson HF, Gelber RD, Rudenstam CM, Goldhirsch A, Senn HJ: **Timing of baseline quality of life assessment in an international adjuvant breast cancer trial: its effects on patient self-estimation. The International Breast Cancer Study Group.** *Ann Oncol* 1994, 5: 65-74.

10. Neises M, Sir MS, Strittmatter HJ, et al: **Influencing of age and of different operative methods on the quality of life in patients with breast cancer.** *Onkologie* 1994, 17: 410-419.

11. Osoba D, Zee B, Pater J, Warr D, Kaizer L, Latreille J: **Psychometric properties and responsiveness of the EORTC quality of life questionnaire (QLQ-C30) in patients with breast, ovarian, and lung cancer.** *Qual Life Res* 1994, 3: 353-364.

12. Shimozuma K, Sonoo H, Ichihara K, Kurebayashi J, Miyake K, Yoshikawa K, Ota K: **Analysis of factors associated with quality of life in breast cancer patients after surgery.** *Breast Cancer* 1994, 1: 123-129.

13. Stefanek ME: **Quality of life and other psychological issues in breast cancer.** *Curr Opin Oncol* 1994, 6: 583-586.

14. Twelves CJ, Dobbs NA, Lawrence MA, Ramirez AJ, Summerhayes M, Richards MA, Towlson KE, Rubens RD: **Iododoxorubicin in advanced breast cancer-A phase II evaluation of clinical activity, pharmachology and quality of life.** *Br J Cancer* 1994, 69: 726-731.

15. Winer EP. **Quality of life research in patients with breast cancer.** *Cancer* 1994, 74 (Suppl. 1): 410-415.

**1993**

1. Aaronson NK: **Assessment of quality of life and benefits from adjuvant therapies in breast cancer.** *Recent Results Cancer Res* 1993, 127, 201-210.

2. Bryson HM, Plosker GL: **Tamoxifen: a review of pharmacoeconomic and quality of life consideration for its use as adjuvant therapy in women with breast cancer.** *Pharmaeconomics* 1993; 4: 40-66.

3. Clavel M, Soukop M, Greenstreet YL: **Improved control of emesis and quality of life with Ondansetron in breast cancer.** *Oncology* 1993, 50: 180-185.

4. Fallowfield L: **Quality of life in breast cancer: results from 3 cancer research campaign studies.** *Acta Clin Belg Suppl* 1993, 15: 19-23.

5. Fallowfield LJ: **Quality of life measurement in breast cancer.** *J Roy Soc Med* 1993, 86: 10-12.

6. Fraser SCA, Dobbs HJ, Ebbes SR, Fallowfield LJ, Bates T, Baum M: **Combination or mild single agent chemotherapy for advanced breast cancer: CMF vs. epirubicin measuring quality of life.** *Br J Cancer* 1993, 67: 402-406.

7. Fraser SCA, Ramirez AJ, Ebbes SR, Fallowfield LJ, Dobbs HJ, Richards MA, Bates T, Baum M: **A daily diary for quality of life measurement in advanced breast cancer trials.** *Br J Cancer* 1993, 67: 341-346.

8. Gelber RD, Cole BF, Goldhirsch A: **How to compare quality of life of breast cancer patients in clinical trials. International Breast Cancer Study Group.** *Recent Results Cancer Res* 1993, 127: 221-233.

9. Gelber RD, Goldhirsch A, Cole BF: **Parametric extrapolation of survival estimates with applications to quality of life evaluation of treatments. International Breast Cancer Study Group.** *Control Clin Trials* 1993, 14: 485-499.

10. Gerard K, Dobson M, Hall J: **Framing and labeling effects in health descriptions: quality adjusted life years for treatment of breast cancer.** *J Clin Epidemiol* 1993, 46: 77-84.

11. Hayden KA, Moinpour CM, Metch B, Feigl P, O’Bryan RM, Green S, Osborne CK: **Pitfalls in quality-of-life assessment: lessons from a Southwest Oncology Group Breast Cancer Clinical Trial.** *Oncol Nurs Forum* 1993, 20: 1415-1419.

12. Kornblith AB, Hollis DR, Zuckerman E, Lyss AP, Canello GP, Cooper MR, Herndon JE, Phillips CA, Abrams J, Aisner J, et al: **Effect of megestrol acetate on quality of life in dose-response trial in women with advanced breast cancer. The Cancer and Leukemia Group B.** *J Clin Oncol* 1993, 11: 2081-2089.

13. Meyerowitz BE: **Quality of life in breast cancer patients: the contribution of data to the care of patients.** *Eur J Cancer* 1993, 29 (Suppl. 1): S59-62.

14. Ovesen L, Hannibal J, Mortensen EL: **The interrelationship of weight loss, dietary intake, and quality of life in ambulatory patients with cancer of the lung, breast, and ovary.** *Nutr Cancer* 1993, 19: 159-167.

15. Wallace LM, Priestman SG, Dunn JA, Priestman TJ: **The quality of life of early breast cancer patients treated by two different radiotherapy regimens.** *Clin Oncol* 1993; 5: 228-233.

16. Wyatt G, Kurtz ME, Liken M: **Breast cancer survivors: an exploration of quality of life issues.** *Cancer Nurs* 1993, 16: 440-448.

17. Yokoe T, Ishida T, Tominaga S, Kuroishi T, Morimoto T, Tashiro H, Itoh S, Abe R, Ota J, Horino T, et al: **Effect of mass screening for breast cancer from the aspect of psychosocial assessment of the quality of life.** *Jap J Cancer Res* 1993, 84: 365-370.

**1992**

1. Campora E, Naso C, Vitullo MT, Giudici S, Camoirano A, Repetto L, Rosso R: **The impact of chemotherapy on the quality of life of breast cancer patients.** *J Chemother* 1992, 4: 59-63.

2. Coates A, Gebski V, Signorini D, Murray P, McNeil D, Byne M, Forbes JF: **Prognostic value of quality of life scores during chemotherapy for advanced breast cancer. Australian New Zealand Breast Cancer Trial Group.** *J Clin Oncol* 1992, 10: 1833-1838.

3. Ganz A, Lee JJ, Sim MS, Polinsky ML, Schag CA: **Exploring the influence of multiple variables on the relationship of age to quality of life in women with breast cancer*.*** *J Clin Epidemiol* 1992, 45: 473-485.

4. Ganz PA, Schag CA, Lee JJ, Polinsy ML, Tan S: **Breast conservation versus mastectomy: is there a difference in psychological adjustment or quality of life in the year after surgery.** *Cancer* 1992, 69: 1729-1738.

5. Gelber RD, Goldhirsch A, Hunry C, Bernhard J, Simes RJ: **Quality of life in clinical trials of adjuvant therapies.** International Breast Cancer Study Group (formerly Ludwig Group). *J Natl Cancer Inst Monogr* 1992, 11: 127-135.

6. Hurny C, Bernhard J, Gelber RD: **Quality of life measures for patients receiving adjuvant therapy for breast cancer: an international trial.** *Eur J Cancer* 1992, 28: 118-124.

7. Richards MA, Hopwood P, Ramirez AJ Twelves CJ, Ferguson J, Gregory WM, Swindell R, Scrivener W, Miller J, Howell A, et al: **Doxorubicin in advanced breast cancer: influence of schedule on response, survival and quality of life.** *Eur J Cancer* 1992, 28: 1023-1028.

8. Soukop M, McQuade B, Hunter E, Stewart A, Kaye S, Cassidy J, Kerr D, Khanna S, Smyth J, Coleman R, et al: **Ondansetron compared with Metoclopramide in the control of emesis: quality of life during repeated chemotherapy for breast cancer.** *Oncology* 1992, 49: 295-304.

**1991**

1. Berglund G, Bolund C, Fornander T, Rutqvist LE, Sjoden PO: **Late effects of adjuvant chemotherapy and postoperative radiotherapy on quality of life among breast cancer.** *Eur J Cancer* 1991, 27: 1075-1081.

2. de Koning HJ, van Ineveld BM, van Oortmarssen GH, de Haes JC, Collett HJ, Hendriks JH, van der Maas PJ: **Breast cancer screening and cost-effectiveness, policy alternatives, quality of life considerations and the possible impact of uncertain factors.** *Int J Cancer* 1991, 49: 531-537.

3. Gelber RD, Goldhirsch A, Cavalli F. **Quality of life adjusted evaluation of adjuvant therapies for operable breast cancer. The International Breast Cancer Study Group.** *Ann Intern Med* 1991, 114: 621-628.

4. Kiebert GM, de Haes JC, van de Velde CJ: **The impact of breast conserving treatment and mastectomy on the quality of life of early stage breast cancer patients: a review.** *J Clin Oncol* 1991, 9: 1059-1070.

5. Tamburini M, Brambilla C, Ferrari L, Bombino T, Gangeri L, Rosso S: **Two simple indexes used to evaluate the impact of therapy on the quality of life of patients receiving primary chemotherapy for operable breast cancer.** *Ann Oncol* 1991, 2; 417-422.

6. van Holten-Verzantvoort AT, Zwinderman AH, Aaronson NK, Hermans J, van Emmerik B, van Dam FS, van den Bos B, Bijvoet OL, Cleton FJ: **The effect of supportive pamidronate treatment on aspects of quality of life of patients with advanced breast cancer.** *Eur J Cancer* 1991, 27: 544-549.

7. Young-McCaughan S, Sexton DL: **A retrospective investigation of the relationship between aerobic exercise and quality of life in women with breast cancer.** *Oncol Nurs Forum* 1991, 18: 751-757.

**1990**

1. Baum M, Ebbs SR, Fallowfield LJ, Fraser SC: **Measurement of quality of life in advanced breast cancer.** *Acta Oncol* 1990, 29: 391-395.

2. Ganz PA, Schag AC, Cheng HL: **Assessing the quality of life: a study in newly diagnosed breast cancer patients.** *J Clin Epidemiol* 1990, 43: 75-86.

3. Ketiku KK, Ajekigbe AT: **Chemotherapy of breast cancer in Nigerians: side-effects and quality of life.** *Clin Oncol* 1990, 2: 153-155.

4. Kiebert GM, Hanneke J, de Haes CJ, Kievit J, van de Velde CJ: **Effect of peri-operative chemotherapy on the quality of life of patients with early breast cancer.** *Eur J Cancer* 1990, 26: 1038-1042.

5. McEvoy MD, McCorkle R. Quality of life issues in patients with disseminated breast cancer. Cancer 1990, 66 (Suppl. 6): 1416-1421.

6. Sutherland HJ, Lockwood GA, Boyd NF: **Ratings of the importance of quality of life variables: therapeutic implications.** *J Clin Epidemiol* 1990, 43; 661-666.

**1989**

1. Brunner KW: **Adjuvant therapies in breast cancer and quality of life: a critical review of the TwiST concept.** *Recent Results Cancer Res* 1989, 115: 239-243.

2. Hurny C: **Critical review of quality of life: psychosocial aspects of adjuvant therapy in breast cancer.** *Recent Results Cancer Res* 1989, 115: 279-282.

**1988**

1. Ciampi A, Lockwood G, Sutherland HJ, Llewellyn-Thomas HA: **Assessment of health related quality of life: factor scales for patients with breast cancer.** *J* *Psychsocial Oncol* 1988, 6: 1-19.

2. Levine MN, Guyatt GH, Gent M, De Pauw S, Goodyear MD, Hryniuk WM, Arnold A, Findlay B, Skillings JR, Bramwell VH, et al: **Quality of life in stage II breast cancer: an instrument for clinical trials.** *J Clin Oncol* 1988, 6: 1798-1810.

**1987**

1. Coates A, Gebski V, Bishop JF, Jeal PN, Woods RL, Snyder R, Tattersall MH, Byrne M, Harvey V, Gill G: **Improving the quality of life during chemotherapy for advanced breast cancer. A comparison of intermittent and continuous treatment strategies.** *N Engl J Med* 1987, 317; 1490-1495.

2. Routh A, Hickman BT: **Quality of life after loco-regional recurrence and in advanced breast cancer*.*** *J Miss State Med Assoc* 1987, 28: 87-90.

**1986**

1. de Haes JC, van Oostrom MA, Welvaart K: **The effect of radical and conserving surgery on the quality of life of early breast cancer patients.** *Eur J Surg Oncol* 1986, 12: 337-342.

**1985**

1. Bell DR, Tannock IF, Boyd NF: **Quality** **of life measurement in breast cancer patients.** *Br J Cancer* 1985, 51: 577-580.

2. de Haes JC, van Oostrom MA, Welvaart K: **Quality of life after breast surgery.** *J* *Surg Oncol* 1985, 28: 123-125.

**1984**

There were no citations.

**1983**

1. Meyerowwitz BE, Watkins IK, Sparks FC: **Quality of life for breast cancer patients receiving adjuvant chemotherapy.** *Am J Nurs* 1983, 83: 232-235. [treatment- adjuvant chemo]

2. Scott DW: **Quality of life following the diagnosis of breast cancer.** *Top Clin Nurs* 1983, 4: 20-37.

**1982**

Morris T: **The impact of the organization of treatment for breast cancer on quality of life.** *Experientia Suppl* 1982, 41: 381-387.

**1981**

There were no citations.

**1980**

1. Palmer BV, Walsh GA, McKinna JA, Greening WP: **Adjuvant chemotherapy for breast cancer: side effects and quality of life.** **Br Med J** 1980, 281: 1594-1597.

**1976**

1. Priestman TJ, Baum M: **Evaluation of quality of life in patients receiving treatment for advanced breast cancer.** *Lancet* 1976, 1: 899-900.

**1974**

1. Moore FD, van de Vanter SB, Boyden CM, Lokich J, Wilson RE: **Adrenalectomy with chemotherapy in the treatment of advanced breast cancer: objective and subjective response rates, duration and quality of life*.*** *Surgery* 1974, 76: 376-390.
